# Incomplete MaxSAT approaches for combinatorial testing

**DOI:** 10.1007/s10732-022-09495-3

**Published:** 2022-08-17

**Authors:** Carlos Ansótegui, Felip Manyà, Jesus Ojeda, Josep M. Salvia, Eduard Torres

**Affiliations:** 1 Logic & Optimization Group (LOG), University of Lleida, Lleida, Spain; 2 Artificial Intelligence Research Institute (IIIA, CSIC), Campus UAB, 08193 Bellaterra, Spain

**Keywords:** Combinatorial testing, Maximum satisfiability, Constraint programming

## Abstract

We present a Satisfiability (SAT)-based approach for building Mixed Covering Arrays with Constraints of minimum length, referred to as the Covering Array Number problem. This problem is central in Combinatorial Testing for the detection of system failures. In particular, we show how to apply Maximum Satisfiability (MaxSAT) technology by describing efficient encodings for different classes of complete and incomplete MaxSAT solvers to compute optimal and suboptimal solutions, respectively. Similarly, we show how to solve through MaxSAT technology a closely related problem, the Tuple Number problem, which we extend to incorporate constraints. For this problem, we additionally provide a new MaxSAT-based incomplete algorithm. The extensive experimental evaluation we carry out on the available Mixed Covering Arrays with Constraints benchmarks and the comparison with state-of-the-art tools confirm the good performance of our approaches.

## Introduction

The Combinatorial Testing (CT) problem (Nie and Leung 2011) addresses the question of how to efficiently verify the proper operation of a system, where a system can be a program, a circuit, a package that integrates several pieces of software, a GUI interface, a cloud application, etc. This problem requires exploring the parameter space of the system by iteratively testing different settings of the parameters to detect errors, bugs or faults. If we consider the system parameters as variables, a setting can be described as a *full* assignment to these parameters.

Exploring all the parameter space exhaustively, i.e., the set of all possible full assignments, is, in general, out of reach. Notice that if a system has a set of parameters *P*, the number of different full assignments is $\prod _{p \in P} g_p = {\mathcal {O}}\left( g^{|P|}\right)$, where $g_p$ is the cardinality of the domain of parameter *p* and *g* is the cardinality of the greatest domain.

The good news is that, in practice, there is no need to explore all the parameter space to detect errors, bugs or faults. We just need to *cover* a portion of the possible parameter combinations (Kuhn et al. 2004). For example, most software errors (75%-80%) are caused by certain individual parameters or by the interaction of just two of them.

To cover that portion of parameter combinations exhaustively, Covering Arrays (CAs) play an important role in CT. Given a set of parameters *P* and a strength *t*, a Covering Array *CA*(*N*; *t*, *P*) is a test suite of *N* tests that guarantee to cover all the possible interactions of *t* parameters (referred as *t*-tuples). Since executing a test in the system has a cost, we are interested in working with relatively small covering arrays. We refer to the minimum *N* for which a *CA*(*N*; *t*, *P*) exists as the Covering Array Number, denoted by *CAN*(*t*, *P*). In particular, we are interested in building an optimal CA, i.e., a covering array of length *CAN*(*t*, *P*). Notice that it is guaranteed that the number of tests required to cover all *t*-way parameter combinations, for fixed *t*, grows logarithmically in the number of parameters (Colbourn 2004), which indicates that optimal or near-optimal covering arrays can be used in practical terms. The computational challenge is to build optimal CAs in a reasonable time frame.

In this paper, we focus on *Mixed* Covering Arrays with *Constraints* (MCACs). The term *Mixed* refers to the possibility of having parameter domains of different sizes. The term *Constraints* refers to the existence of some parameter interactions that are not allowed in the system. These forbidden interactions are usually implicitly described by a set of constraints. The problem of computing an MCAC of minimum length, to which we refer in this paper as the Covering Array Number problem, is NP-hard (Maltais and Moura 2010).

There exist several greedy approaches that tackle the problem of building minimum MCACs, such as PICT (Czerwonka 2006), based on the OTAT framework (Bryce et al. 2005), and ACTS (Borazjany et al. 2012), based on the IPOG algorithm (Duan et al. 2017). One downside of these approaches is that they become more inefficient as the hardness of the set of forbidden interactions increases. Therefore, we are more interested in constraint programming approaches, which are better suited for handling constraints. For example, CALOT (Yamada et al. 2015) is a tool for building MCACs based on Satisfiability (SAT) technology (Biere et al. 2009) that can handle constraints efficiently.

Within constraint programming techniques (Rossi et al. 2006), SAT technology provides a highly competitive generic problem approach for solving decision problems. In particular, the decision problem to be solved is translated into a SAT instance (a propositional formula) and a SAT solver is used to determine whether there is a solution. In this paper, we will *review* in detail the CALOT tool, which essentially solves a sequence of SAT instances to compute an optimal MCAC. Each SAT instance in the sequence encodes the decision query of whether there exists an MCAC of a certain length. By iteratively bounding the length, the optimum can be determined.

Since the problem of computing minimum MCACs is, in essence, an optimization problem, we also consider its reformulation into the Maximum Satisfiability (MaxSAT) problem (Biere et al. 2009), which is an optimization version of the SAT problem.

We show empirically that MaxSAT approaches outperform ACTS and CALOT (the state-of-the-art) once the suitable MaxSAT encodings are used. We evaluate both complete or exact MaxSAT solvers (certify optimality) and incomplete MaxSAT solvers (provide suboptimal solutions). In particular, we show that while complete MaxSAT solvers perform similar to CALOT (substantially in contrast to previously reported experiments with MaxSAT solvers (Yamada et al. 2015)), incomplete MaxSAT solvers obtain better suboptimal solutions and faster than ACTS and CALOT on many instances. This confirms the practical interest of incomplete MaxSAT approaches because, in real environments, we are mainly concerned with obtaining the best possible solution within a given budget of runtime.

Having confirmed the good performance of MaxSAT approaches for computing minimum MCACs, we explore another related problem, the Tuple Number (TN) Problem. Informally, the TN problem is to determine the minimum set of missing *t*-tuples in a test suite of *N* tests, or the maximum set of *t*-tuples that these *N* tests cover. This problem is related to the Optimal Shortening Covering Arrays (OSCAR) problem (Carrizales-Turrubiates et al. 2011) (which is NP-hard), where given a matrix of tests the goal is to find a submatrix of a fixed number of tests and parameters that maximizes the number of covered *t*-tuples. These *shortened* covering arrays have been used to improve the initialization of metaheuristic approaches for Covering Arrays (without SUT constraints).

In this paper, we explore (for the first time) the *Mixed* and *with Constraints* variants of the TN problem, assessing the performance of complete and incomplete MaxSAT approaches. Obviously, this problem is of interest when $N < CAN(t,P)$.^1^ We additionally present another incomplete approach based on MaxSAT technology to which we refer as MaxSAT Incremental Test Suite (Maxsat ITS), that *incrementally* builds the test suite with the help of a MaxSAT query that aims to maximize the coverage of allowed tuples at every step.

The Covering Array Number problem is concerned with reporting solutions with the least number of tests. From a practical point of view, whether we are satisfied with suboptimal solutions will depend on the cost of the tests. This cost basically includes the cost of generating the tests (computational resources) and the cost of testing the system. In particular, when the cost is too prohibitive in terms of our budget, and we are satisfied with covering a statistically significant portion of the tuples, we aim to solve (even suboptimally) the Tuple Number problem. Therefore, there exist real-world scenarios where all the approaches described in this paper are of practical interest.

The rest of the paper is structured as follows: Sect. 2 introduces definitions on CAs, SAT/MaxSAT instances, constraints and SAT solvers. For computing MCACs of a given length, Sect. 3 defines different SAT encodings and Sects. 4 and 5 describe techniques to make the SAT encodings more efficient. Section 6 introduces the incremental SAT algorithm CALOT for computing minimum MCACs. Subsequently, Sect. 7 defines MaxSAT encodings and Sect. 8 describes how to efficiently apply MaxSAT solvers. For the Tuple Number problem, Sect. 9 defines a MaxSAT encoding and Sect. 10 presents a new incomplete approach using MaxSAT solvers. To assess the impact of the presented approaches, Sect. 11 reports on an extensive experimental investigation on the available MCAC benchmarks. Finally, Sect. 12 concludes the paper.

## Preliminaries

We first introduce the definitions related to Systems Under Test and Covering Arrays.

### Definition 1

A System Under Test (SUT) model is a tuple $\langle P,\varphi \rangle$, where *P* is a finite set of variables *p* of finite domain, called SUT parameters, and $\varphi$ is a set of constraints on *P*, called SUT constraints, that implicitly represents the parameterizations that the system accepts. We denote by *d*(*p*) and $g_p$, respectively, the domain and the domain cardinality of *p*. For the sake of clarity, we will assume that the system accepts at least one parameterization.

In the following, we assume $S=\langle P,\varphi \rangle$ to be a SUT model. We will refer to *P* as $S_P$, and to $\varphi$ as $S_{\varphi }$.

### Example 1

As an example of SUT model, we focus on the domain of autonomous driving. Table 1 shows the parameters and values, $S_P$, and the SUT constraints, $S_\varphi$:

**Table 1:** Example of autonomous driving system under test

$P \in S_P$	Abbrv.	Values
Luminosity	L	Day (dy), Night (ni)
Environment	E	Highway (hw), Urban (ur), Country (co)
Motor	M	Combustion (cb), Electric (el)
Sensor	S	Camera (ca), Radar (ra), Lidar (li)
$$S_\varphi $$		
$$((L = ni) \wedge (E = co)) \rightarrow (S \ne ca)$$		
$$((E = hw) \vee (E = co)) \rightarrow (S \ne li)$$		
$$(M = el) \rightarrow (E = ur)$$		

### Definition 2

An assignment is a set of pairs (*p*, *v*) where *p* is a variable and *v* is a value of the domain of *p*. A test case for *S* is a full assignment *A* to the variables in $S_P$ such that *A* entails $S_\varphi$ (i.e. $A \models S_\varphi$) . A parameter tuple of *S* is a subset $\pi \subseteq S_P$. A value tuple of *S* is a partial assignment to $S_P$; in particular, we refer to a value tuple of length *t* as a *t*-tuple.

### Example 2

Consider the SUT presented in Example 1. An example of test case is $\{(L,dy),(E,hw),(S,ca),(M,cb)\}$.

$\{L, E\}$ is a parameter tuple and $\{(L,dy),(E,hw)\}$ a value tuple for $t=2$.

### Definition 3

A *t*-tuple $\tau$ is forbidden if $\tau$ does not entail $S_\varphi$ (i.e. $\tau \models \lnot S_\varphi$). Otherwise, it is allowed. We refer to the set of allowed *t*-tuples as $\mathcal {T}_a^{t,S} = \{\tau \ |\ \tau \not \models \lnot S_\varphi \}$, to the set of forbidden *t*-tuples as $\mathcal {T}_f^{t,S} = \{\tau \ |\ \tau \models \lnot S_\varphi \}$, and to the whole set of *t*-tuples in the SUT model *S* as $\mathcal {T}^{t,S} = \mathcal {T}_a \cup \mathcal {T}_f$.

When there is no ambiguity, we refer to $\mathcal {T}_a^{t,S},\mathcal {T}_f^{t,S},\mathcal {T}^{t,S}$ as $\mathcal {T}_a,\mathcal {T}_f,\mathcal {T}$, respectively.

### Example 3

The total number of *t*-tuples $|\mathcal {T}|$ for $t=2$ in the SUT of Example 1 is 37. The set of forbidden tuples $\mathcal {T}_f$ is:

$$\begin{aligned} \mathcal {T}_f = \{\{(E,co), (M, el)\},\{(E, hw),(S,li)\},\{(E,hw),(M,el)\},\{(E,co),(S,li)\}\} \end{aligned}$$

Therefore, the number of allowed tuples $|\mathcal {T}_a|$ is 33.

### Definition 4

A test case $\upsilon$ *covers* a value tuple $\tau$ if both assign the same domain value to the variables in the value tuple, i.e., $\upsilon \models \tau$.

### Example 4

In Example 1, the test case $\upsilon = \{(L,dy),(E,hw),(S,ca),(M,cb)\}$ covers the following *t*-tuples for $t=2$:

$$\begin{aligned}{} & {} \{(L,dy),(E,hw)\}, \{(L,dy),(S,ca)\}, \{(L,dy),(M,cb)\}, \\{} & {} \{(E,hw),(S,ca)\}, \{(E,hw),(M,cb)\}, \{(S,ca),(M,cb)\} \end{aligned}$$

### Definition 5

A Mixed Covering Array with Constraints (MCAC), denoted by *CA*(*N*; *t*, *S*), is a set of *N* test cases for a SUT model *S* such that all *t*-tuples are at least covered by one test case. We refer to parameter *t* as the *strength* of the Covering Array.

The term *Mixed* reflects that the domains of the parameters in $S_P$ are allowed to have different cardinalities.

The term *Constraints* reflects that $S_\varphi$ is not empty.

### Example 5

An example of MCAC for the SUT in Example 1 is shown in Table 2:

**Table 2:** *CA*(10; 2, *S*) for the autonomous driving SUT

	L	E	S	M
$\upsilon _1$	dy	hw	ca	cb
$\upsilon _2$	ni	hw	ra	cb
$\upsilon _3$	ni	ur	ca	el
$\upsilon _4$	dy	ur	ra	cb
$\upsilon _5$	dy	ur	li	cb
$\upsilon _6$	dy	co	ca	cb
$\upsilon _7$	ni	co	ra	cb
$\upsilon _8$	dy	ur	ra	el
$\upsilon _9$	ni	ur	li	cb
$\upsilon _{10}$	ni	ur	li	el

### Definition 6

The Covering Array Number, *CAN*(*t*, *S*), is the minimum *N* for which there exists an MCAC *CA*(*N*; *t*, *S*). An upper bound $ub^{CAN(t,S)}$ for *CAN*(*t*, *S*) is an integer such that $ub^{CAN(t,S)} \ge CAN(t,S)$, and a lower bound $lb^{CAN(t,S)}$ is an integer such that $CAN(t,S) > lb^{CAN(t,S)}$.

When there is no ambiguity, we refer to $ub^{CAN(t,S)}$ ($lb^{CAN(t,S)}$) as *ub* (*lb*).

### Example 6

Given the SUT in Example 1, $CAN(2, S) = 8$ as shown in Table 3.

**Table 3:** *CA*(8; 2, *S*) for the autonomous driving SUT. This corresponds to the *CAN*(2, *S*)

	L	E	S	M
$\upsilon _1$	dy	ur	el	ra
$\upsilon _2$	dy	hw	cb	ca
$\upsilon _3$	dy	ur	cb	li
$\upsilon _4$	dy	co	cb	ca
$\upsilon _5$	ni	hw	cb	ra
$\upsilon _6$	ni	ur	el	ca
$\upsilon _7$	ni	ur	el	li
$\upsilon _8$	ni	co	cb	ra

### Definition 7

The Tuple Number, *T*(*N*; *t*, *S*), is the maximum number of *t*-tuples that can be covered by a set of *N* tests for a SUT model *S*. An upper bound $ub^{T(N;t,S)}$ for *T*(*N*; *t*, *S*) is an integer such that $ub^{T(N;t,S)} \ge T(N;t,S)$, and a lower bound $lb^{T(N;t,S)}$ is an integer such that $T(N;t,S) > lb^{T(N;t,S)}$.

When there is no ambiguity, we refer to $ub^{T(N;t,S)}$ ($lb^{T(N;t,S)}$) as *ub* (*lb*).

### Example 7

Given the SUT model in Example 1, $T(8;2,S) = 33$ since $CAN(2, S) = 8$ and there are exactly 4 forbidden 2-tuples. Additionally, $T(7;2,S) = 31$ and $T(6;2,S) = 29$.

### Definition 8

The MCAC problem is to find an MCAC of size *N*.

The Covering Array Number problem is to find an MCAC of size *CAN*(*t*, *S*).

The Tuple Number problem is to find a test suite of size *N* that covers *T*(*N*; *t*, *S*) *t*-tuples.

The MCAC problem is a decision problem. The Covering Array Number and the Tuple Number problems, to which we refer in short as the *CAN*(*t*, *S*) and *T*(*N*; *t*, *S*) problems, respectively, are optimization problems.

Now, we introduce the definitions related to the encodings and the SATisfiability-based solving technology we will use to solve the problems defined above.

### Definition 9

A literal is a propositional variable *x* or a negated propositional variable $\lnot x$. A clause is a disjunction of literals. A formula in Conjunctive Normal Form (CNF) is a conjunction of clauses.

### Definition 10

A weighted clause is a pair (*c*, *w*), where *c* is a clause and *w*, its weight, is a natural number or infinity. A clause is hard if its weight is infinity (or no weight is given); otherwise, it is soft. A Weighted Partial MaxSAT instance is a multiset of weighted clauses.

### Example 8

The following Weighted Partial MaxSAT instance $\phi = \{(x_1, 2), (x_2, 1), (x_1 \vee x_2, 3)$, $(\lnot x_1 \vee \lnot x_2, \infty )\}$ contains exactly 3 soft clauses and 1 hard clause.

### Definition 11

A truth assignment for an instance $\phi$ is a mapping that assigns to each propositional variable in $\phi$ either 0 (False) or 1 (True). A truth assignment is *partial* if the mapping is not defined for all the propositional variables in $\phi$.

### Definition 12

A truth assignment *I* satisfies a literal *x* $(\lnot x)$ if *I* maps *x* to 1 (0). A truth assignment *I* falsifies a literal *x* $(\lnot x)$ if *I* maps *x* to 0 (1). A truth assignment *I* satisfies a clause if *I* satisfies at least one of its literals; otherwise, it is violated or falsified. The cost of a clause (*c*, *w*) under *I* is 0 if *I* satisfies the clause; otherwise, it is *w*. Given a partial truth assignment *I*, a literal or a clause is undefined if it is neither satisfied nor falsified. A clause *c* is a unit clause under *I* if *c* is not satisfied by *I* and contains exactly one undefined literal.

### Definition 13

The cost of a formula $\phi$ under a truth assignment *I*, denoted by $cost(I, \phi )$, is the aggregated cost of all its clauses under *I*.

### Example 9

Given $I=\{x_1 = 0, x_2 = 0\}$ and the instance $\phi$ in Example 8, the $cost(I, \phi )$ is 6.

### Definition 14

The Weighted Partial MaxSAT (WPMaxSAT) problem for an instance $\phi$ is to find an assignment in which the sum of weights of the falsified soft clauses is minimal (referred to as the optimal cost of $\phi$) and all the hard clauses are satisfied. The Partial MaxSAT problem is the WPMaxSAT problem when all the soft clauses have the same weight. The MaxSAT problem is the Partial MaxSAT problem when there are no hard clauses. The SAT problem is the Partial MaxSAT problem when there are no soft clauses.

### Example 10

The optimal cost of the instance $\phi$ in Example 8 is 1.

### Definition 15

An instance of Weighted Partial MaxSAT, or any of its variants, is unsatisfiable if its optimal cost is $\infty$. A SAT instance $\varphi$ is satisfiable if there is a truth assignment *I*, called model, such that $cost(I, \varphi ) = 0$.

### Definition 16

An unsatisfiable core is a subset of clauses of a SAT instance that is unsatisfiable.

### Definition 17

Given a SAT instance $\varphi$ and a partial truth assignment *I*, we refer as Unit Propagation, denoted by $UP(I,\varphi )$, to the Boolean inference mechanism (propagator) defined as follows: Find a unit clause in $\varphi$ under *I*, where *l* is the undefined literal. Then, propagate the unit clause, i.e. extend *I* with $x=1$ ($x=0$) if $l \equiv x$ ($l \equiv \lnot x$) and repeat the process until a fixpoint is reached or a conflict is derived (i.e. a clause in $\phi$ is falsified by *I*).

We refer to $UP(I,\varphi )$ simply as $UP(\varphi )$ when *I* is empty.

### Example 11

Given the SAT instance $\varphi = \{(x_1 \vee x_2), (x_1 \vee \lnot x_2 \vee x_3 \vee x_4)\}$ and the partial truth assignment $I = \{x_1 = 0\}$, $UP(I,\varphi )$ simplifies $\varphi$ to $\{(x_3 \vee x_4)\}$ and extends *I* to $\{x_1 = 0, x_2 = 1\}$.

### Definition 18

Let *A* and *B* be SAT instances.

$A \models B$ denotes that *A* entails *B*, i.e. all assignments satisfying *A* also satisfy *B*.

It holds that $A \models B$ iff $A \wedge \lnot B$ is unsatisfiable.

$A \vdash _{UP} B$ denotes that, for every clause $c \in B$, $UP(A \wedge \lnot c)$ derives a conflict.

If $$A \vdash _{UP} B$$ then $$A \models B$$.

### Definition 19

A *pseudo-Boolean* (PB) constraint is a Boolean function of the form $\sum _{i=1}^{n} q_il_i \diamond k$, where *k* and the $q_i$ are integer constants, $l_i$ are literals, and $\diamond \in \{<, \le , =, \ge , >\}$.

### Definition 20

A Cardinality (Card) constraint is a PB constraint where all $q_i$ are equal to 1. An *At-Most-One* (AMO) constraint is a cardinality constraint of the form $\sum _{i=1}^{n} l_i \le 1$. An *At-Least-One* (ALO) constraint is a cardinality constraint of the form $\sum _{i=1}^{n} l_i \ge 1$. An *Exactly-One* (EO) constraint is a cardinality constraint of the form $\sum _{i=1}^{n} l_i=1$.


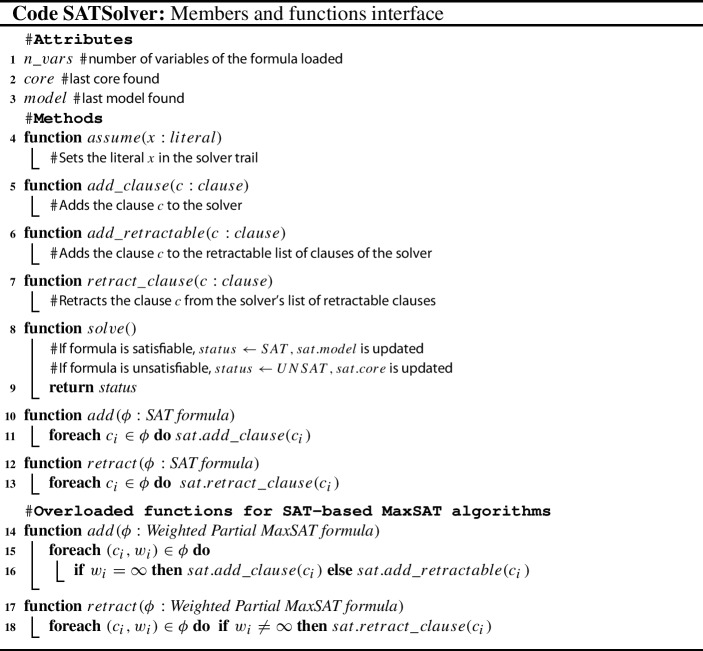


The interface of a modern SAT solver is presented in code fragment SATSolver. The input instance is added to the solver with functions $add\_clause$ and $add\_retractable$ (in case the clause can be retracted) (lines 5-7), which operate on a single clause, while functions *add* and *retract* operate on a set of clauses. The last two functions are overloaded to ease the usage of SAT solvers within MaxSAT solvers (lines 10-13 and 14-18). Variable $n\_vars$ indicates the number of variables of the input formula (line 1).

Function *solve* (lines 8-9) returns UNSAT (SAT) if the input formula is unsatisfiable (satisfiable) and sets variable *core* (*model*) to the corresponding unsatisfiable core (model). Function *assume* (line 4) allows to place an *assumption* on the truth value of a literal before function *solve* is called. Finally, modern SAT solvers also support an incremental solving mode, which allows to keep the learnt clauses across calls to the function *solve*.

## The MCAC problem as SAT

In this section, we present the SAT encoding described in Yamada et al. (2015) to decide whether there exists a *CA*(*N*; *t*, *S*) for a given SUT model $S=\langle P,\varphi \rangle$. It is similar to previous encodings described in Hnich et al. (2005, 2006); Banbara et al. (2010); Nanba et al. (2012); Ansótegui et al. (2013).

In the following, we list the set of constraints that define the SAT encoding and describe the semantics of the propositional variables they refer to. To encode each constraint, we assume that AMO and EO cardinality constraints are translated into CNF through the regular encoding (Ansótegui and Manyà 2004; Gent and Nightingale 2004) and the typical transformations (Tseitin 1983) of $\rightarrow$ and $\leftrightarrow$ are implicitly applied.^2^

First, we define variables $x_{i,p,v}$ to be true iff test case *i* assigns value *v* to parameter *p*, and state that each parameter in each test case takes exactly one value as follows (where $[N] = \{1,\ldots ,N\}$): 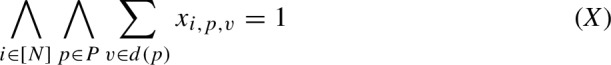


Second, as described in Nanba et al. (2012), to enforce the SUT constraints $\varphi$, for each test case *i*, we add the CNF formula that encodes the constraints of $\varphi$ into SAT and substitute each appearance of the pair (*p*, *v*) in $\varphi$ by the corresponding literal on the propositional variable $x_{i,p,v}$ for each test case *i*. 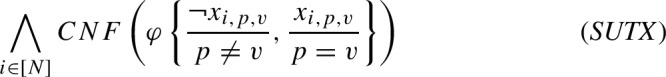


Third, we introduce propositional variables $c_{\tau }^{i}$ and state that if they are true, then tuple $\tau$ must be covered at test *i*, by forcing the variables *p* in the test case to be assigned to the value specified in $\tau$, as follows: 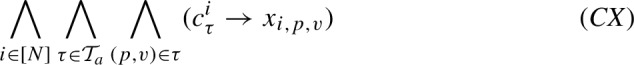


Notice that only *t*-tuples that can be covered by a test case are encoded, i.e., $\tau \in {\mathcal {T}}_a$. In Sect. 4, we discuss how to detect the *t*-tuples forbidden by the SUT constraints.

Finally, we state that every *t*-tuple $\tau \in \mathcal {T}_a$, must be covered at least by one test case, as follows: 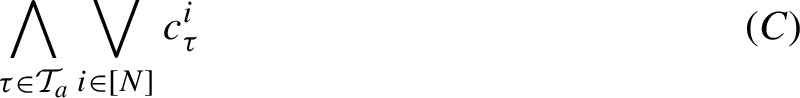


### Proposition 1

Let $Sat_{CX}^{N,t,S}$ be $X \wedge C \wedge CX \wedge SUTX$. $Sat_{CX}^{N,t,S}$ is satisfiable iff a *CA*(*N*; *t*, *S*) exists.

Inspired by the incremental SAT approach in Yamada et al. (2015) (see Sect. 6), we present another encoding where *C* and *CX* are replaced by *CCX*: 
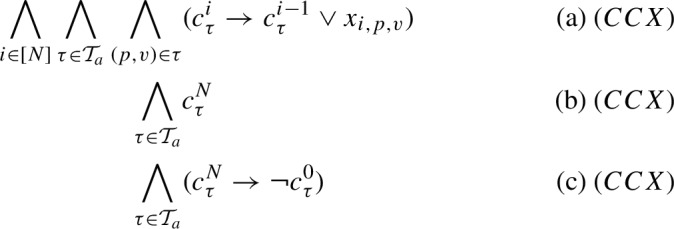


Variables $c^i_{\tau }$ have now a different semantics, i.e., if they are true, $\tau$ is covered by test case *i* or by any lower test case *j*, where $1 \le j \le i$ (equation a). In order to guarantee that $\tau$ will be covered by some test, notice that we just need to force $c^{N}_{\tau }$ to be true and $c^{0}_{\tau }$ to be false (variables $c^{0}_{\tau }$ are additionally included in the encoding). This can be achieved by adding the unit clauses $c^{N}_{\tau }$ (equation b) and the implication $c^{N}_{\tau } \rightarrow \lnot c^{0}_{\tau }$ (equation c) for every allowed tuple $\tau$.

The seasoned reader may wonder why we do not simply replace equation (c) by $\bigwedge _{\tau \in \mathcal {T}_a} \lnot c^{0}_{\tau }$. Indeed, this is possible. First, notice that UP on the conjunction of equations (b) and (c) will derive exactly the same. Second, for encoding some problems where it is not mandatory to cover all the tuples (see Sect. 9 on encoding the Tuple Number problem), we have to erase equation (b) from *CCX* and also guarantee that if a tuple $\tau$ is not covered in an optimal solution, i.e., $c^{N}_{\tau }$ has to be False, then the related clauses in *CCX* have to be satisfied (these are hard clauses) and, if possible, to be trivially satisfied, i.e., without requiring search. Equation (c) eases this case for all the scenarios in Sect. 9. Notice that, once $c^{N}_{\tau }$ is False, clauses in equation (c) are trivially satisfied and, by setting the remaining $c^{i}_\tau$ vars to True, clauses in equation (a) are also trivially satisfied.

### Proposition 2

Let $Sat_{CCX}^{N,t,S}$ be $X \wedge CCX \wedge SUTX$. $Sat_{CCX}^{N,t,S}$ is satisfiable iff a *CA*(*N*; *t*, *S*) exists.

### Remark 1

There are some variations of equation (a) in *CCX* that can be beneficial when using some SAT solvers, as we will see in Sect. 11.1. For example, we can use full implication instead of half implication in equation (a), i.e., $(c^{i}_{\tau } \leftrightarrow c^{i-1}_{\tau } \vee x_{i,p,v})$, or we can even use $(c^{i}_{\tau } \rightarrow c^{i-1}_{\tau } \vee x_{i,p,v}) \wedge (c^{i}_{\tau } \leftarrow c^{i-1}_{\tau })$. Also, we can consider full implication in equation (c) and, for some of the problems analyzed in Sect. 11.1, we can even replace equation (c) by $\bigwedge _{\tau \in \mathcal {T}_a} \lnot c^{0}_{\tau }$.

### Example 12

We show how to build $Sat_{CX}^{N=10,t=2,S}$ for the SUT in Example 1, where $N=10$ is an upper bound *ub* for this SUT (see Sect. 4).

To encode the *X* constraint, we add:Next, for each test $(1,\ldots ,10)$, we encode the SUT constraints *SUTX*:

Ex. SUTX $$\begin{aligned} (x_{1,L,ni} \wedge x_{1,E,co}) \rightarrow \lnot x_{1,S,ca} \\ (x_{1,E,hw} \vee x_{1,E,co}) \rightarrow \lnot x_{1,S,li} \\ x_{1,M,el} \rightarrow x_{1,E,ur} \\ \vdots \\ (x_{10,L,ni} \wedge x_{10,E,co}) \rightarrow \lnot x_{10,S,ca} \\ (x_{10,E,hw} \vee x_{10,E,co}) \rightarrow \lnot x_{10,S,li} \\ x_{10,M,el} \rightarrow x_{10,E,ur} \end{aligned}$$

Finally, the encoding of the *CX* and *C* constraints is shown below. We identify the set of allowed tuples, ($\mathcal {T}_a$), as described in Sect. 4. In particular, there are $|\mathcal {T}_a|=33$ allowed tuples as we mention in Example 3.

Ex. CX $$\begin{aligned} c_{\tau _1}^1 \rightarrow x_{1,L,dy}, \quad \ldots , \quad c_{\tau _{33}}^1 \rightarrow x_{1,M,el} \\ c_{\tau _1}^1 \rightarrow x_{1,E,hw}, \quad \ldots , \quad c_{\tau _{33}}^1 \rightarrow x_{1,S,li} \\ \vdots \qquad \qquad \ddots \qquad \qquad \vdots \\ c_{\tau _1}^{10} \rightarrow x_{10,L,dy}, \quad \ldots , \quad c_{\tau _{33}}^{10} \rightarrow x_{10,M,el} \\ c_{\tau _1}^{10} \rightarrow x_{10,E,hw}, \quad \ldots , \quad c_{\tau _{33}}^{10} \rightarrow x_{10,S,li} \end{aligned}$$

Ex. C $$\begin{aligned} (c_{\tau _1}^1 \vee c_{\tau _1}^2 \vee \cdots \vee c_{\tau _1}^{10}), \quad \ldots , \quad (c_{\tau _{33}}^1 \vee c_{\tau _{33}}^2 \vee \cdots \vee c_{\tau _{33}}^{10}) \end{aligned}$$

To build $Sat_{CCX}^{N=10,t=2,S}$, we encode the *CCX* constraint instead of the *C* and *CX* constraints:

Ex. CCX b $$\begin{aligned} c_{\tau _1}^{10}, \quad \ldots , \quad c_{\tau _{33}}^{10} \end{aligned}$$

Ex. CCX c $$\begin{aligned} c_{\tau _1}^{10} \rightarrow \lnot c_{\tau _1}^0, \quad \ldots , \quad c_{\tau _{33}}^{10} \rightarrow \lnot c_{\tau _{33}}^0 \end{aligned}$$

Once we run a SAT solver on any of the previous SAT instances, if there exists a *CA*(10; 2, *S*), it will return a satisfying truth assignment. To recover the particular *CA*(10; 2, *S*) implicitly found by the solver, we just need to check the assignment to the $x_{i,p,v}$ variables. For example, if $x_{1,L,dy}$ is True then parameter *Luminosity* takes value *day* at test 1.

## Preprocessing for the MCAC problem

In the context of the Covering Array Number problem, we define an upper bound *ub* and a lower bound *lb* to be integers such that $ub \ge CAN(t,S) > lb$. When $ub = lb +1$, we can stop the search and report *ub* as the minimum covering array number *CAN*(*t*, *S*).

To get an initial value for *ub*, we can execute a greedy approach to obtain a suboptimal *CA*(*N*; *t*, *S*) and set *ub* to *N*. For example, in the experiments, we use the tool ACTS (Borazjany et al. 2012) that supports Mixed Covering Arrays with Constraints. Moreover, a lower *ub* also implies a smaller initial encoding.

Additionally, by inspecting the solution, i.e., the test cases that certify the suboptimal *CA*(*N*; *t*, *S*), we can compute which tuples are not covered, the set of *forbidden* tuples, since the suboptimal *CA*(*N*; *t*, *S*) guarantees to cover all allowed *t*-tuples.

Furthermore, let *r* be the maximum number of allowed *t*-tuples associated with any parameter tuple of length *t*. Then, we can set $lb = r-1$, since these *r* value tuples (mutually exclusive) need to be covered by different test cases.
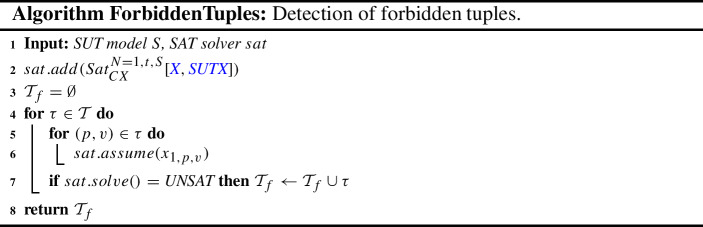


Using an approach like ACTS, not based on constraint programming techniques, has a drawback. It may not be efficient enough if testing the satisfiability of $S_\varphi$ (the set of SUT constraints) is computationally hard. In this case, to detect the forbidden tuples, we can simply apply algorithm ForbiddenTuples. This algorithm tests, for every tuple $\tau$ (lines 4-7), if it is compatible with the SUT constraints (line 2) through a SAT query; if the solver results in unsatisfiability (line 7), the tuple is added to the set of forbidden tuples $\mathcal {T}_f$, which is ultimately returned by the algorithm (line 8).

For $t=2$, which is already of practical importance (Kuhn et al. 2004), the experiments carried out in this paper show that this detection process is negligible runtime-wise.

## Symmetry breaking for the MCAC problem

As Yamada et al. (2015), we fix the *r* *t*-tuples that conducted us to set the initial *lb* (see Sect. 4) to test cases $\{1,\ldots ,r\}$. This helps us break row symmetries for the first *r* test cases. We will refer to this as fixed-tuple symmetry breaking.

There are other alternatives. We can impose row symmetry breaking constraints as Flener et al. (2002); since each row (test) represents a number in base 2, we can add constraints to order the tests in monotonic increasing order, from test 0 to test $N-1$. We can also apply, as explained above, fixed-tuple symmetry breaking to the first *r* tuples (first partition) and apply row symmetry breaking constraints to the remaining $ub-lb+1$ test cases (second partition). Furthermore, we can impose an order among the tuples in the first partition and the second partition, so that if two sets share the same value for the fixed tuple, then the one representing the lower number must be in the first partition.

Our experimental analysis shows that fixed-tuple symmetry breaking is superior to any other of the mentioned alternatives. For lack of space, we restricted all the experiments to this symmetry breaking approach.

### Example 13

We show how to apply symmetry breaking to the SUT in Example 1.

(*E*, *S*) is the parameter tuple with the largest number of allowed tuples we selected. Its set of allowed value tuples is: $\{\tau _1 = \{(E,hw),(S,ca)\}$, $\tau _2 = \{(E,hw),(S,ra)\}$, $\tau _3 = \{(E,ur),(S,ca)\}$, $\tau _4 = \{(E,ur),(S,ra)\}$, $\tau _5 = \{(E,ur),(S,li)\}$, $\tau _6 = \{(E,co),(S,ca)\}, \tau _7 = \{(E,co),(S,ra)\}\}$.

To apply the fixed-tuple symmetry breaking variant, we just need to fix each allowed value tuple in a different test as shown below:

Ex. SYM X $$\begin{aligned} x_{1,E,hw} \wedge x_{1,S,ca} \\ x_{2,E,hw} \wedge x_{2,S,ra} \\ x_{3,E,ur} \wedge x_{3,S,ca} \\ x_{4,E,ur} \wedge x_{4,S,ra} \\ x_{5,E,ur} \wedge x_{5,S,li} \\ x_{6,E,co} \wedge x_{6,S,ca} \\ x_{7,E,co} \wedge x_{7,S,ra} \end{aligned}$$

## Solving the *CAN*(*t*, *S*) problem with Incremental SAT

In this section, we present the CALOT algorithm, which is an incremental SAT approach for computing optimal covering arrays with SUT constraints described by Yamada et al. (2015). The input to the algorithm is an upper bound *ub* (computed as in Sect. 4), the strength *t* and the SUT model *S*. In line 2, the incremental SAT solver is initialized with the SAT instance $Sat_{CCX}^{N=ub,t,S}$. Additionally, breaking symmetries for the first $lb +1$ tuples, as described in Sect. 5, are added to the SAT solver. The output is the covering array number and an optimal model.
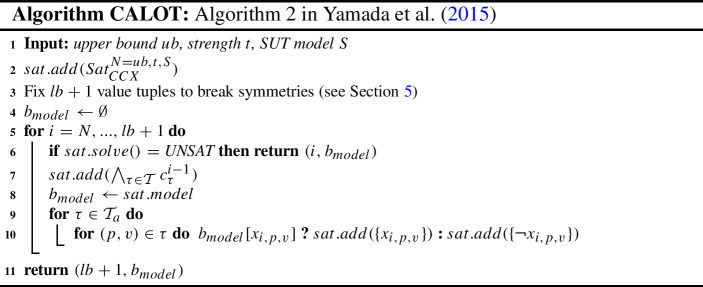


The algorithm works by iteratively decreasing the *ub* till it reaches $lb+1$ (line 5) or the current SAT instance is unsatisfiable (line 6). To decrease the *ub* by one, the algorithm adds the set of unit clauses $\bigwedge _{\tau \in \mathcal {T}_a} c^{i-1}_{\tau }$ (line 7), which state that every *t*-tuple is covered by a test case with an index smaller than *i*.

There is a subtle detail in lines 9 and 10. Whenever the algorithm finds a new upper bound, variables $x_{i,p,v}$ related to the previous upper bound are fixed to the value in the last model found ($b_{model}$ in line 8), so that these variables do not need to be decided in the next iterations. As Yamada et al. (2015) report, not fixing these variables can have some negative impact on the performance.

### Remark 2

The original (Yamada et al. 2015)’s algorithm pseudocode is slightly different. First, it assigns the *i*-th test at iteration *i* to the value it had in the previous model found instead of assigning the $i+1$-th test. This does not correspond to the description given in the text of the paper and may lead to an incomplete algorithm.

Second, the set of constraints (a) (*CCX*), described in Yamada et al. (2015), does not set $c^{N}_{\tau }$ to True as we do in this paper, which makes the pseudocode perform a dummy first step that can cause to report a wrong optimum. We think that these are merely errors in the description, and we have fixed them. Since the tool CALOT is not available from the authors for reproducibility, we have tried to do our best to reproduce (or extend) the idea behind their work.

In Sect. 8, we will see that this SAT incremental approach resembles how SAT-based MaxSAT algorithms behave (Ansótegui et al. 2013; Morgado et al. 2013). Actually, in contrast to Yamada et al. (2015), we show that MaxSAT technology can be effectively applied to solve Covering Arrays.

## The *CAN*(*t*, *S*) problem as partial MaxSAT

Ansótegui et al. (2013) proposes an encoding into Partial MaxSAT to build covering arrays without constraints of minimum size. The main idea is to use an indicator variable $u_i$ that is True iff test case *i* is used to build the covering array. The objective function of the optimization problem, which aims to minimize the number of variables $u_i$ set to True, is encoded into Partial MaxSAT by adding the following set of soft clauses: 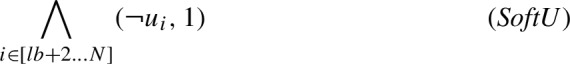


Notice that we only need to use $N-(lb+1)$ indicator variables since we know that the covering array will have at least $lb+1$ tests (see Sect. 4).

To avoid symmetries, it is also enforced that if test case $i+1$ belongs to the minimum covering array, so does the previous test case *i*: 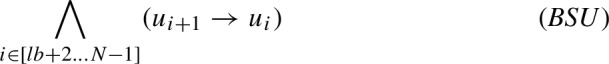


Then, variables $u_i$ are connected to variables $c^i_{\tau }$, expressing that if we want test *i* to be the proof that $\tau$ is covered, then test *i* must be in the optimal solution^3^: 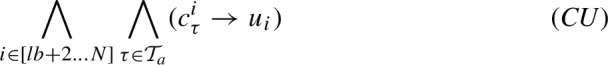


### Proposition 3

Let $PMSat_{CX}^{N,t,S,lb}$ be $SoftU \wedge BSU \wedge CU \wedge Sat_{CX}^{N,t,S}$. If $N \ge CAN(t,S)$, the optimal cost of the Partial MaxSAT instance $PMSat_{CX}^{N,t,S,lb}$ is $CAN(t,S)-(lb+1)$, otherwise it is $\infty$.

In order to build the Partial MaxSAT version of $Sat_{CCX}^{N,t,S}$, we just need to change how variables $u_i$ are related to variables $c^{i}_{\tau }$. This constraint reflects that if $u_i$ is False (i.e., test *i* is not in the solution and, therefore, due to constraint *BSU*, none of the tests $>i$ cannot be in the solution either), then the tuple $\tau$ has to be covered at some test below *i*: 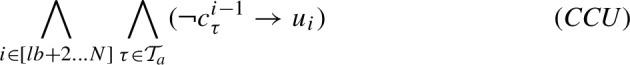


### Proposition 4

Let $PMSat_{CCX}^{N,t,S,lb}$ be $SoftU \wedge BSU \wedge CCU \wedge Sat_{CCX}^{N,t,S}$. If $N \ge CAN(t,S)$, the optimal cost of the Partial MaxSAT instance $PMSat_{CCX}^{N,t,S,lb}$ is $CAN(t,S)-(lb+1)$, otherwise it is $\infty$.

### Remark 3

In Ansótegui et al. (2013), variables $u_i$ are instead connected to variables $x_{i,p,v}$ in the following way: 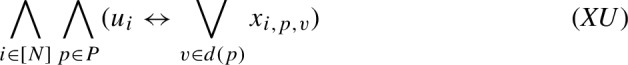


This is a more compact encoding but it requires Eq. *X* to use an AMO constraint instead of an EO constraint.

Finally, we can convert these Partial MaxSAT instances into Weighted Partial MaxSAT modifying *SoftU* as follows: 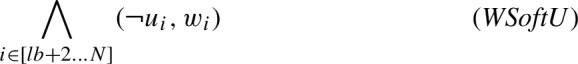 If we use $w_i = 2^{i-(lb+2)}$ we naturally introduce a lexicographical preference in the soft constraints. This is a key detail to alter the behaviour of SAT-based MaxSAT algorithms when solving Covering Arrays. If the MaxSAT solver applies the stratified approach (Ansótegui et al. 2012) (see for more details Sect. 8), it suffices to use $w_i = i-(lb+2)+1$, i.e., to increase the weights linearly. This is of interest since a high number of tests in *WSoftU* can result in too large weights for some MaxSAT solvers.

### Proposition 5

Let $$WPMSat_{CCX}^{N,t,S,lb}$$ be $$WSoftU \wedge BSU \wedge CCU \wedge Sat_{CCX}^{N,t,S}$$.

If $N \ge CAN(t,S)$ and $w_i = 2^{i-(lb+2)}$ the optimal cost of the Weighted Partial MaxSAT instance $WPMSat_{CCX}^{N,t,S,lb}$ is $2^{CAN(t,S) - (lb + 1)} -1$, otherwise it is $\infty$.

If $N \ge CAN(t,S)$ and $w_i = i-(lb+2)+1$ the optimal cost of the Weighted Partial MaxSAT instance $WPMSat_{CCX}^{N,t,S,lb}$ is $(1+n) \cdot n/2$ where $n=CAN(t,S) - (lb + 1)$, otherwise it is $\infty$.

### Example 14

We extend our working example to obtain the Partial MaxSAT and Weighted Partial MaxSAT encodings described in this section. We first describe how we encode *SoftU* (left) and *BSU* (right) constraints:

Ex. SoftU and BSU $$\begin{aligned} \begin{array}{c} (\lnot u_{10}, 1) \\ (\lnot u_9, 1) \\ (\lnot u_8, 1) \end{array}{} & {} \begin{array}{c} u_{10} \rightarrow u_9 \\ u_9 \rightarrow u_8 \end{array} \end{aligned}$$

Recall that in our example $ub=10$ and $lb=6$ (see Examples 12 and 13). Therefore, we will have $N - (lb + 1) = 10 - (6 + 1) = 3$ $u_i$ indicator variables.

To build the $PMSat_{CX}^{N=10,t=2,S,lb=6}$ instance we add to $Sat_{CX}^{N=10,t=2,S}$ the *CU* constraint:

Ex. CU $$\begin{aligned} c_{\tau _1}^{10} \rightarrow u_{10}, \quad \ldots , \quad c_{\tau _{33}}^{10} \rightarrow u_{10} \\ c_{\tau _1}^9 \rightarrow u_9, \quad \ldots , \quad c_{\tau _{33}}^9 \rightarrow u_9 \\ c_{\tau _1}^8 \rightarrow u_8, \quad \ldots , \quad c_{\tau _{33}}^8 \rightarrow u_8 \end{aligned}$$

To build $PMSat_{CCX}^{N=10,t=2,S,lb=6}$ we add to $Sat_{CCX}^{N=10,t=2,S}$ the *CCU* constraint:

Ex. CCU $$\begin{aligned} \lnot c_{\tau _1}^{9} \rightarrow u_{10}, \quad \ldots , \quad \lnot c_{\tau _{33}}^{9} \rightarrow u_{10} \\ \lnot c_{\tau _1}^8 \rightarrow u_9, \quad \ldots , \quad \lnot c_{\tau _{33}}^8 \rightarrow u_9 \\ \lnot c_{\tau _1}^7 \rightarrow u_8, \quad \ldots , \quad \lnot c_{\tau _{33}}^7 \rightarrow u_8 \end{aligned}$$

The weighted counterparts, $WPMSat_{CX}^{N=10,t=2,S,lb=6}$ and $WPMSat_{CCX}^{N=10,t=2,S,lb=6}$, need only to replace *SoftU* by *WSoftU* (using $w_i = i-(lb+2)+1$), as follows:

Ex. WSoftU $$\begin{aligned} (\lnot u_{10}, 3) \\ (\lnot u_9, 2) \\ (\lnot u_8, 1) \end{aligned}$$

To build the resulting MCAC from the MaxSAT solver truth assignment, we will discard the $x_{i,p,v}$ vars whose corresponding $u_i$ is assigned to False (i.e. test *i* does not belong to the solution), and proceed as in Example 12.

## Solving the *CAN*(*t*, *S*) problem with MaxSAT

In this section, we show that SAT-based MaxSAT approaches can *simulate*^4^ the CALOT algorithm, while the opposite is not true. This is an interesting insight since the MaxSAT approach additionally provides the option of applying a plethora of MaxSAT algorithms.

Let us first introduce a short description of SAT-based MaxSAT algorithms. For further details, please consult (Ansótegui et al. 2013; Morgado et al. 2013). Roughly speaking, SAT-based MaxSAT algorithms proceed by reformulating the MaxSAT optimization problem into a sequence of SAT decision problems. Each SAT instance of the sequence encodes whether there exists an assignment to the MaxSAT instance with a cost less than or equal to a certain *k*. SAT instances with a *k* less than the optimal cost are unsatisfiable, while the others are satisfiable. The SAT solver is executed in incremental mode to keep the clauses learnt at each iteration over the sequence of SAT instances. Thus, SAT-based MaxSAT can also be viewed as a particular application of incremental SAT solving.

There are two main types of SAT-based MaxSAT solvers: (i) model-guided and (ii) core-guided. The first ones iteratively refine (decrease) the upper bound and guide the search with satisfying assignments (models) obtained from satisfiable SAT instances. The second ones iteratively refine (increase) the lower bound and guide the search with the unsatisfiable cores obtained from unsatisfiable SAT instances. Both have strengths and weaknesses, and hybrid approaches exist (Ansótegui et al. 2016; Ansótegui and Gabàs 2017).

### The linear MaxSAT algorithm

The Linear algorithm (Eén and Sörensson 2006; Le Berre and Parrain 2010), described in Algorithm Linear, is a model-guided algorithm for WPMaxSAT. Let $\phi = \phi _s \cup \phi _h$ (line 1) be the input WPMaxSAT instance, where $\phi _s$ ($\phi _h$) is the set of soft (hard) clauses in $\phi$.
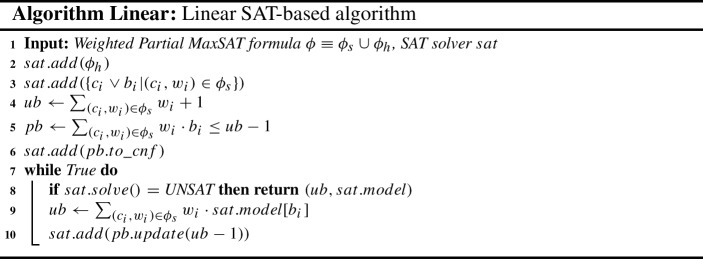


At each iteration of the Linear algorithm, the SAT instance solved by the incremental SAT solver is composed of: (i) the hard clauses $\phi _h$ (line 2), which guarantee that any possible solution is a *feasible* solution; (ii) the reification of each soft clause $(c_i,w_i) \in \phi _s$ into clause $(c_i \vee b_i)$, where $b_i$ is a fresh auxiliary variable which acts as a collector of the truth value of the soft clause (line 3); and (iii) the CNF translation of the PB constraint $\sum _{(c_i,w_i) \in \phi _s} w_i \cdot b_i \le k$, where $k = ub-1$ bounds the aggregated cost of the falsified soft clauses, i.e., the value of the objective function.

Initially, *ub* is set to $(\sum _{(c_i,w_i) \in \phi _s} w_i + 1)$ (line 4), that is semantically equivalent to $\infty$. Then, iteratively, if the incremental SAT solver returns satisfiable, *ub* is updated to $(\sum _{(c_i,w_i) \in \phi _s} w \cdot sat.model[b_i])$ (line 9)^5^; otherwise, *ub* is the optimal cost (line 8). If the input instance is unsatisfiable the algorithm returns $\sum _{(c_i,w_i) \in \phi _s} w_i + 1$ (i.e., $\infty$).

A technical point to mention is that the PB constraint is translated into SAT thanks to an incremental PB encoding (line 5) so that whenever we tighten the upper bound, instead of retracting the original PB constraint and encode the new one, we just need to add some additional clauses (line 10). Additionally, if all the weights in the soft clauses are equal, instead of using an incremental PB encoding, we can use an incremental cardinality encoding for which more efficient encodings do exist.

#### Proposition 6

The Linear algorithm with Weighted Partial MaxSAT instance $WPMSat_{CCX}^{N,t,S,lb}$ as input can *simulate* the CALOT algorithm (excluding lines 9 and 10).

In the first place notice that in the worst case the Linear algorithm will decrease the current upper bound by one unit as the Algorithm CALOT. Then, the key point establishing the connection of the Linear algorithm with the CALOT algorithm is to show that, given the same upper bound *k* to both algorithms, the Linear algorithm can propagate the same set of $c^{i-1}_{\tau }$ variables (line 7 in Algorithm CALOT).

Let us recall that the Linear algorithm, with input $\phi \equiv WPMSat_{CCX}^{N,t,S,lb}$, will generate a sequence of SAT instances composed of the original hard clauses $\phi _h$, the reification of the soft clauses $\bigwedge _{(c_i,w_i) \in \phi _s} (c_i \vee b_i)$, the translation to CNF of the PB constraint $\sum _{(c_i,w_i) \in \phi _s} w_i \cdot b_i \le k$, where $(c_i,w_i)$ represents the *i*-th soft clause in $WPMSat_{CCX}^{N,t,S,lb}$, i.e., $(\lnot u_i, 2^{i-(lb+2)})$ when using the exponential increase, and the current upper bound *k*.

#### Proposition 7

If $$\phi \equiv WPMSat_{CCX}^{N,t,S,lb}$$, then

$$\begin{aligned}{} & {} CCU \wedge \bigwedge _{(\lnot u_i, 2^{i-(lb+2)}) \in \phi _s} (\lnot u_i \vee b_i) \wedge \sum _{(\lnot u_i, 2^{i-(lb+2)}) \in \phi _s} 2^{i-(lb+2)} \cdot b_i \le k \vdash _{UP} \\{} & {} \quad \bigwedge _{k < i \le N+1} \bigwedge _{\tau \in \mathcal {T}_a} c^{i-1}_{\tau }. \end{aligned}$$

First of all, notice that the weight of a higher index test is strictly greater than the aggregated weights of the lower index tests. Given an upper bound *k*, an *efficient* CNF translation of the PB constraint will allow Unit Propagation (UP) to derive that all *b*s associated with soft clauses with a weight greater than *k* must be False. Then, from the set of clauses that reify the soft clauses (of the form $\lnot u_i \vee b_i$), UP will also derive that the corresponding $u_i$ vars must be False and, from the set of hard clauses *CCU*, UP will derive that the corresponding $c^{i-1}_{\tau }$ must be true.

If the input problem is a Partial MaxSAT instance, i.e., $PMSat_{CCX}^{N,t,S,lb}$ where the *i*-th soft clause is of the form $(\lnot u_i,1)$, the Linear algorithm uses a cardinality constraint instead of a PB constraint to bound the aggregated cost of the falsified soft clauses. In this case, we can only guarantee that $CCU \wedge \bigwedge _{(\lnot u_i,1) \in \phi _s} (\lnot u_i \vee b_i) \wedge \sum _{(\lnot u_i,1) \in \phi _s} b_i \le k \models \bigwedge _{k < i \le N+1} \bigwedge _{\tau \in \mathcal {T}_a} c^{i-1}_{\tau }$. Notice that, given an upper bound *k*, UP cannot derive on $\sum _{(\lnot u_i,1) \in \phi _s} b_i \le k$ the set of $b_i$s that must be False, because all correspond to soft clauses of equal weight.

**CALOT algorithm cannot** ***simulate*** **the Linear Algorithm** While the CALOT algorithm decreases the upper bound by one at each iteration, the Linear algorithm can decrease it more aggressively. This is the case when it finds a model with a lower cost than $k-1$ (line 9), which can significantly reduce the number of calls to the SAT solver.

### The WPM1 MaxSAT algorithm

The Fu &Malik algorithm (Fu and Malik 2006) is a core-guided SAT-based MaxSAT algorithm for Partial MaxSAT instances. In contrast to the Linear algorithm, which uses the models to iteratively refine the upper bound, the Fu &Malik algorithm uses the unsatisfiable cores to refine the lower bound. In particular, the initial SAT instance $\varphi _0$ explored by the Fu &Malik algorithm is composed of the hard clauses in the input MaxSAT instance $\phi _h$ plus the SAT clauses $c_i$ *extracted* from the soft clauses $(c_i,w_i)$. We refer to these $c_i$ clauses as soft-indicator clauses.

At each iteration, if $\varphi _k$ is satisfiable, the optimum is *k*. If $\varphi _k$ is unsatisfiable, the clauses in the unsatisfiable core retrieved by the SAT solver are analyzed. If none of the clauses is a soft-indicator clause, the Partial MaxSAT formula is declared unsatisfiable and the algorithm stops. Otherwise, the core tells us that we need to relax the soft-indicator clauses, i.e., we need to violate more clauses. To construct the next instance, $\varphi _{k+1}$, each soft-indicator clause in the core of $\varphi _{k}$ is relaxed with a fresh auxiliary variable *b* and a hard EO cardinality constraint is added on these new variables, indicating that at least one clause must be violated (this is what the core told us) and at most one clause is violated (this prevents jumping over the optimum).

**The WPM1 algorithm** (Ansótegui et al. 2009; Manquinho et al. 2009) is an extension of the Fu &Malik algorithm that solves Weighted Partial MaxSAT instances by applying the split rule for weighted clauses. In particular, we are interested in using the *Stratified* WPM1 algorithm (WPM1) (Ansótegui et al. 2012), which clusters the input clauses according to their weights.^6^ These clusters were originally named as strata in Ansótegui et al. (2012). The algorithm incrementally merges the clusters solving the related subproblem until all clusters have been merged. In its simpler version, all the clauses in a cluster have the same weight (called the representative weight), and clusters are added in decreasing order with respect to the representative weight, but other strategies can also be applied (Ansótegui et al. 2012).
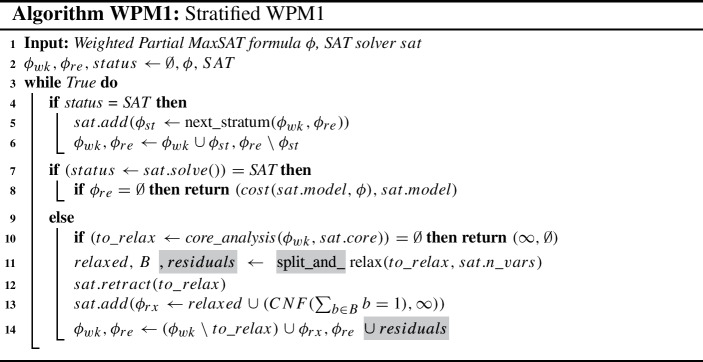


In the WPM1 algorithm, variable $\phi _{wk}$ represents the formula that contains the merged clusters (strata) so far, while $\phi _{re}$ represents the remaining weighted clauses from the original input instance $\phi$. Whenever we solve to optimality the current instance $\phi _{wk}$, i.e., the SAT solver returned a SAT answer in the last call (line 4) but $\phi _{re} \ne \emptyset$, function $next\_stratum$ updates variable $\phi _{st}$ to the new stratum (cluster) to be merged with $\phi _{wk}$^7^ (the working SAT instance (line 5) and variables $\phi _{wk}, \phi _{re}$ are updated accordingly (line 6)). Otherwise, the SAT solver returned UNSAT in the previous call, meaning that we are still optimizing the current subproblem $\phi _{wk}$ and need to call the SAT solver again (line 7).

If the SAT solver returns a SAT answer and all the original clauses in $\phi$ have been considered, i.e. $\phi _{re} = \emptyset$, then we have optimized the input instance $\phi$ and return its cost and an optimal model (line 8).

If the SAT solver returns an UNSAT answer, first we analyze the unsatisfiable core returned by the SAT solver (line 10) and return the soft-indicator clauses to be relaxed in variable $to\_relax$, if any; otherwise, we have certified that the set of hard clauses is unsatisfiable, i.e., we return cost $\infty$ and an empty model.

Function $split\_and\_relax$ (line 11) first applies the split rule to the soft-indicator clauses in $to\_relax$ and generates two sets, one where all the clauses are normalized to have the minimum weight, and another with the residuals of each clause with respect to the minimum weight in $to\_relax$. Second, the set of clauses with the minimum weight are extended, each with an additional fresh variable and stored in the set *relaxed* as in the Fu &Malik algorithm. The new fresh variables are returned in set *B*.

Finally, the original set of clauses $to\_relax$ is retracted from the SAT solver (line 12), and the new set *relaxed* is added to the working SAT instance plus the cardinality constraint that increases the lower bound as in the Fu &Malik algorithm (line 13).^8^ In line 14, $\phi _{wk}$ is updated to reflect the changes in the SAT working formula, and the remaining formula $\phi _{re}$ is extended with the residuals generated from the application of the split rule.

As a final remark, notice that if the statements in grey boxes of the WPM1 algorithm are erased and function $next\_stratum$ is instructed to report sequentially, first the hard clauses and then the soft clauses, we get the original Fu &Malik algorithm.

In the context of the Covering Array Number problem, the Fu &Malik algorithm on the $PMSat_{CCX}^{N,t,S,lb}$ instance will perform a bottom-up search, i.e, the first query will correspond to the question of whether the covering array can be constructed with $k=0$ tests, then with $k=1$ tests, etc. This approach does not provide any intermediate upper bounds since the only query answered positively corresponds to the optimum.

However, interestingly, by considering the weighted version of the Fu &Malik algorithm, we can perform a top-down search on the Covering Array problem and provide intermediate upper bounds.

#### Proposition 8

The Stratified WPM1 algorithm with input $WPMSat_{CCX}^{N,t,S,lb}$ can *simulate* the CALOT algorithm (excluding lines 9 and 10).

Back to the context of covering arrays, each cluster in $WPMSat_{CCX}^{N,t,S,lb}$ would be composed of a single soft clause $(\lnot u_i, w_i)$, except the cluster containing all the hard clauses. The first subproblem seen by the Stratified WPM1 algorithm encodes the query of whether one can build a covering array using *N* tests. The next subproblem incorporates the first soft clause $(\lnot u_N, w_N)$ and encodes the query of whether one can construct the covering array using $N-1$ tests. Notice that each $\lnot u_i$ will propagate, according to *CCU*, the corresponding $c^{i-1}_{\tau }$ vars as in the CALOT algorithm. Notice also that every solution of a subproblem is an upper bound for the covering array.

The discussion of this section has provided insights into how to solve Covering Arrays through MaxSAT, but also into how to fix similar difficulties in other problems where MaxSAT is not yet effective enough.

### Test-based Streamliners for the *CAN*(*t*, *S*) problem

Notice that a solution for a *CAN*(*t*, *S*) problem can be extended to multiple solutions in the previous MaxSAT translations. This happens when $CAN(t,S) < N$, since the assignment to the *x* vars related to any test *i* with $i>CAN(t,S)$ (useless from the point of view of the *CAN*(*t*, *S*) problem) still needs to be consistent with the *X* and *SUTX* constraints. In general, notice that *SUTX* can be NP-complete.

Lines 9 and 10 of the CALOT algorithm, as described in Sect. 6, fix that problem but cannot directly be applied within MaxSAT algorithms since the solver is not aware of the *CAN*(*t*, *S*) problem semantics.

However, we can reproduce a similar effect. At the preprocessing step, we can build a *dummy* test case $\upsilon$ by computing a solution to $S_\varphi$ (e.g. with a SAT solver) or select any of the test cases in the solution returned by the ACTS tool when computing the upper bound (see Sect. 4). Then, we can state in the MaxSAT encoding that if a given test *i* is not part of the optimal solution (i.e., $u_i$ is False), then the corresponding *x* vars are set to the value in the test case $\upsilon$. 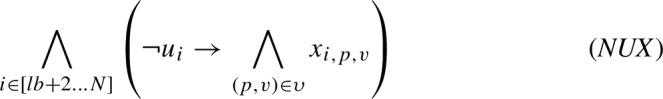


The *dummy* test case $\upsilon$ exactly plays the role of the so-called streamliner constraints (Gomes and Sellmann 2004), which rule out some of the possible solutions but make the search of the remaining solutions more efficient.

There is yet another way to mitigate that potential bottleneck. We can indeed extend *SUTX* clauses for test *i* with literal $\lnot u_i$. Therefore, whenever test *i* is no longer in the optimal solution (i.e. $u_i$ is False), the corresponding *SUT* constraints are trivially satisfied. However, in the experimental investigation, we confirmed that this option is less efficient than adding *NUX* clauses.

#### Example 15

For the SUT in Example 1, let us assume that we use the following *dummy* test $\upsilon = \{(L,dy),(E,ur),(S,ra),(M,cb)\}$. Then, the *NUX* encoding for $\upsilon$ is:

Ex. NUX $$\begin{aligned} \lnot u_{10} \rightarrow (x_{10,L,dy} \wedge x_{10,E,ur} \wedge x_{10,S,ra} \wedge x_{10,M,cb}) \\ \lnot u_{9} \rightarrow (x_{9,L,dy} \wedge x_{9,E,ur} \wedge x_{9,S,ra} \wedge x_{9,M,cb}) \\ \lnot u_{8} \rightarrow (x_{8,L,dy} \wedge x_{8,E,ur} \wedge x_{8,S,ra} \wedge x_{8,M,cb}) \end{aligned}$$

## The *T*(*N*; *t*, *S*) problem as weighted partial MaxSAT

For some applications, we may not be able to use as many test cases as the covering array number (e.g. due to budget restrictions), but we may still be interested in solving the Tuple Number problem, i.e., to determine the maximum number of covered *t*-tuples we can get with a test suite of fixed size.

Once again, MaxSAT technology can play an important role when SUT constraints are considered. Moreover, the size of the SAT/MaxSAT encodings for this problem are smaller than encodings for computing the Covering Array Number, since fewer tests are taken into consideration.

In the following, we show how we can modify the $Sat_{CX}^{N,t,S}$ and $Sat_{CCX}^{N,t,S}$ formulae to become Partial MaxSAT encodings of the Tuple Number problem.

The basic idea is that we need to soften the hard restriction that enforces all allowed *t*-tuples to be covered. To this end, we modify the SAT instance $Sat_{CX}^{N,t,S}$ as follows: First, we soften all the clauses from Eq. *C* which encode that every *t*-tuple $\tau$ must be covered by at least one test case, therefore allowing to violate (or relax) these constraints. For the sake of clarity, although not required for soundness, we introduce a new set of indicator variables $c_{\tau }$ that reify each ALO constraint in Eq. *C* by introducing the following hard constraints: 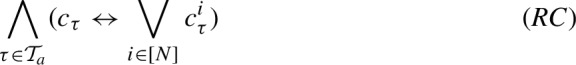


Then, we add the following set of soft clauses: 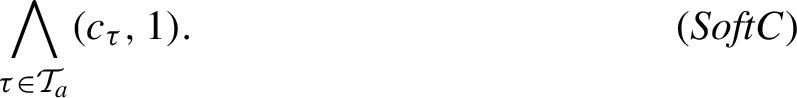


Finally, we we replace in $Sat_{CX}^{N,t,S}$ the set of constraints *C* (the hard constraint that forced to cover all the tuples) by the previous two sets of constraints.

### Proposition 9

Let *S* be a SUT model and let $TPMSat_{CX}^{N,t,S}$ be $Sat_{CX}^{N,t,S}\left\{ \frac{SoftC \wedge RC}{C}\right\}$. The optimal cost of $TPMSat_{CX}^{N,t,S}$ is $|\mathcal {T}_a| - T(N;t,S)$.

### Remark 4

Even if $N>lb$, we cannot use fixed-tuple symmetry breaking since we do not know whether the *t*-tuples that we fix will lead to an optimal solution. Therefore, fixed-tuple symmetry is disabled for all the encodings in this section.

### Remark 5

When computing the tuple number, we can avoid the step of detecting all forbidden tuples since the encoding remains sound, i.e., we can interchange $\mathcal {T}_a$ by $\mathcal {T}$. Notice that those $c_{\tau }$ vars related to forbidden tuples will always be set to False. Moreover, notice that a core-guided algorithm may potentially detect easily as many unsatisfiable cores as forbidden tuples which include just the unit soft clause that represents the forbidden tuple.

In case we want to extend $Sat_{CCX}^{N,t,S}$ to compute the tuple number, we just need to notice that the previously defined role of $c_{\tau }$ corresponds exactly to variable $c^{N}_{\tau }$ in $Sat_{CCX}^{N,t,S}$, so we just need to soften the hard unit clauses $c^{N}_{\tau }$ (described in *CCX*) with weight 1.

### Proposition 10

Let *S* be a SUT model and let $TPMSat_{CCX}^{N,t,S}$ be $Sat_{CCX}^{N,t,S}\left\{ \frac{(c^{N}_{\tau },1)}{(c^{N}_{\tau }) }\right\}$. The optimal cost of $TPMSat_{CCX}^{N,t,S}$ is $|\mathcal {T}_a| - T(N;t,S)$.

### Example 16

We show how to build $TPMSat_{CX}^{N=10,t=2,S}$ for the SUT in Example 1.

We must create a new variable $c_{\tau }$ for each value tuple in $\mathcal {T}_a$ and then replace constraint *C* in $SAT_{CX}^{N=10,t=2,S}$ (see Example 12) by *RC* (left). Finally, we have to add the *SoftC* soft clauses (right):

Ex. RC and SoftC $$\begin{aligned} \begin{array}{c} c_{\tau _1} \leftrightarrow (c_{\tau _1}^1 \vee c_{\tau _1}^2 \vee \cdots \vee c_{\tau _1}^{10}) \\ \vdots \\ c_{\tau _{33}} \leftrightarrow (c_{\tau _{33}}^1 \vee c_{\tau _{33}}^2 \vee \cdots \vee c_{\tau _{33}}^{10}) \end{array}{} & {} \begin{array}{c} (c_{\tau _1}, 1) \\ \vdots \nonumber \\ (c_{\tau _{33}}, 1) \end{array} \end{aligned}$$

For the $TPMSat_{CXX}^{N=10,t=2,S}$, we just have to soften, with weight 1, the set of clauses from *CCX* (*b*) in $SAT_{CCX}^{N=10,t=2,S}$ (see Example 12).

In what follows, we present two extensions.

### Combining the *CAN*(*t*, *S*) and *T*(*N*; *t*, *S*) problems

The Covering Array and Tuple Number problems can lead to thinking about a more general formulation of the optimization problem where we want to maximize the number of covered *t*-tuples while minimizing the number of test cases. Notice that it will depend on the value of *N* with respect to the covering array number (not necessarily known a priori) whether we are, in essence, solving the covering array number or the tuple number problem.

To this end, we take the $PMSat_{CX}^{N,t,S,lb}$ encoding of the Covering Array Number problem for a SUT model *S*, *N* tests and strength *t*. As earlier shown in this section, we first replace the set of hard constraints *C* by *RC* and *SoftCWU*. 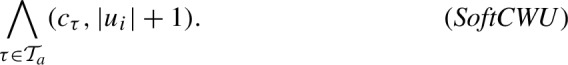


Notice that we prefer violating all soft clauses $(\lnot u_i,1)$ over violating a single soft clause $(c_{\tau }, |u_i|+1)$. This way, we guarantee that any solution to our new Weighted Partial MaxSAT instance maximises the number of covered *t*-tuples while minimises the number of needed test cases.

#### Proposition 11

If $N \ge CAN(t,S)$, the optimal cost of the Weighted Partial MaxSAT instance $PMSat_{CX}^{N,t,S,lb}\left\{ \frac{SoftCWU \wedge RC }{C}\right\}$ is $CAN(t,S) - (lb + 1) + (|\mathcal {T}_a| - T(N;t,S)) \cdot (|u_i|+1)$, otherwise it is $N - (lb+1) + (|\mathcal {T}_a| - T(N;t,S)) \cdot (|u_i|+1)$.^9^

The same idea can be applied to $PMSat_{CCX}^{N,t,S,lb}$ by softening the unit hard clauses $(c^{N}_{\tau })$ in equation (b) from *CCX* with weight $|u_i|+1$. Here, it is important to recall the discussion in Sect. 3 on the need of equation (c) in *CCX*. The other, perhaps more natural, alternative was to replace equation (c) in *CCX* by $\bigwedge _{\tau \in \mathcal {T}_a} \lnot c^{0}_{\tau }$. The problem arises when, in an optimal solution, $\tau$ is not covered, what also implies that $(c^{N}_{\tau })$ is False. Notice that we need to satisfy all clauses related to $\tau$ in *CCX* but, in order to do that, we need to set all $c^{i}_{\tau }$ vars to False. This may not be compatible with equation *CCU* (clauses of the form $\lnot c^{i-1}_{\tau } \rightarrow u_i$) when some test *i* is discarded to be in the solution and variable $u_i$ is set to False, since UP will derive in *CCU* that $c^{i-1}_{\tau }$ is True. In this case, a contradiction is reached. On the other hand, as discussed in Sect. 3, equation (c) allows to set all $c^{i}_{\tau }$ vars to True when $(c^{N}_{\tau })$ is False and trivially satisfy all clauses in *CCX* related to $\tau$.

#### Proposition 12

If $N \ge CAN(t,S)$, the optimal cost of the Weighted Partial MaxSAT instance $PMSat_{CCX}^{N,t,S,lb}\left\{ \frac{SoftCWU }{(c^{N}_{\tau })}\right\}$ is $CAN(t,S) - (lb + 1) + (|\mathcal {T}_a| - T(N;t,S)) \cdot (|u_i|+1)$, otherwise it is $N - (lb+1) + (|\mathcal {T}_a| - T(N;t,S)) \cdot (|u_i|+1)$. ^9^

### The *CAN*(*t*, *S*) problem with relaxed tuple ratio coverage as MaxSAT

We can tackle other realistic settings where we still want to use the minimum number of tests, but there is no need to achieve a 100% ratio of covered *t*-tuples (mandatory per definition in Covering Arrays). Notice that the last tests that shape the covering array number tend to cover very few not yet covered *t*-tuples. Therefore, if these tests are expensive enough in our setting, we may consider relaxing the ratio coverage and skip these tests.

The mentioned problem can be encoded by replacing the previously soft constraints on the $c_{\tau }$ vars with a hard cardinality constraint on the minimum number of *t*-tuples to be covered as follows: 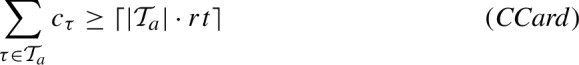


where *rt* is the ratio of allowed *t*-tuples that we want to cover. Notice that, for efficiency reasons, *CCard* can be also described as $\sum _{\tau \in \mathcal {T}_a} \lnot c_{\tau } \le \lceil |\mathcal {T}_a| \cdot (1-rt)\rceil$.

#### Remark 6

With this formulation, we cannot use the fixed-tuples symmetry breaking since we do not know whether we will require at least *lb* tests to cover the specified ratio of allowed *t*-tuples.

#### Proposition 13

Let $RTPMSat_{CCX}^{N,t,S,rt}$ be $PMSat_{CCX}^{N,t,S,lb=0}\left\{ \frac{CCard}{(c^{N}_{\tau })}\right\}$. The optimal cost of $RTPMSat_{CCX}^{N,t,S,rt}$ is the minimum $N'$ such that $T(N',t,S) \ge \lceil |\mathcal {T}_a| \cdot rt \rceil$.

## Incomplete MaxSAT algorithms for the *T*(*N*; *t*, *S*) problem

As argued earlier, if certifying optimality is not a requirement and we are just interested in obtaining a good suboptimal solution in a reasonable amount of time, we can apply incomplete MaxSAT algorithms on the encodings of the Tuple Number problem described in the previous section. Additionally, in this section, we present a new incomplete algorithm to compute suboptimal solutions for the Tuple Number problem.

### MaxSAT based incremental test suite construction

A way to reduce the search space of any constraint problem is to add the so-called streamliner constraints (Gomes and Sellmann 2004). We recall that these constraints rule out some of the possible solutions but make the search for the remaining solutions more efficient. However, in practice, streamliners can rule out all the solutions.

In our context, the streamliner constraints correspond to a set of tests that we think have the potential to be part of optimal solutions. By fixing these tests, we generate a new covering array problem, easier to solve, but whose Covering Array Number can be greater than or equal to that of the original covering array, because we may have missed all the optimal solutions. We iterate this process until all *t*-tuples get covered. To select the *k* candidate test to be fixed at each iteration, we solve the Tuple Number problem restricted to length *k*.

In the context of the Tuple Number problem, this iterative process of fixing tests should not only finish when all *t*-tuples have been covered but also when the requested *N* tests have been fixed.

To that end, here we combine a greedy iterative approach with the SAT-based MaxSAT approaches from Sect. 9 in the IncrementalCA algorithm.
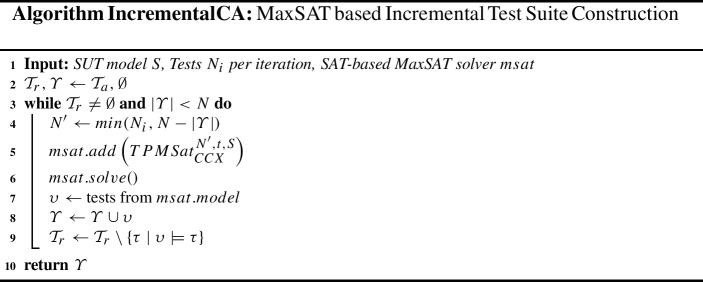


In this algorithm, we begin with the remaining tuples to cover $\mathcal {T}_r$, initially assigned to allowed tuples $\mathcal {T}_a$, as well as an empty test suite $\varUpsilon$ (line 2). Then, we first check how many tests should be encoded; the minimum between the tests in iteration $N_i$ and the remaining number of tests left to complete the test suite, $N-|\varUpsilon |$ (line 4), storing the result into $N^\prime$. Next, we solve the Tuple Number problem for these $N^\prime$ tests, encoded as a $TPMSat_{CCX}^{N^\prime ,t,S}$ formula (lines 5, 6) from Sect. 9. We extract the model from the MaxSAT solver, interpreting it into newly found test cases $\upsilon$ (line 7). Then, those new tests are added to test suite $\varUpsilon$ (line 8). Finally, the tuples covered by these new test cases are removed from $\mathcal {T}_r$ (line 9). This iteration is repeated until no more tuples are left in $\mathcal {T}_r$ or we have reached the requested *N* test cases (line 3), in which case we return the constructed test suite $\varUpsilon$ (line 10).

## Experimental evaluation

In this section, we report on an extensive experimental investigation conducted to assess the approaches proposed in the preceding sections. We start by defining the benchmarks, which include 28 industrial, real-world or real-life instances and 30 crafted instances, and the algorithms involved in the evaluation.

We contacted the authors of Yamada et al. (2015) and Yamada et al. (2016) to obtain the benchmarks used in their experiments. In particular, the available benchmarks are: (i) Cohen et al. (2008), with 5 real-world and 30 artificially generated (crafted) covering array problems; (ii) Segall et al. (2011), with 20 industrial instances; (iii) Yu et al. (2015), with two real-life systems reported by ACTS users; and (iv) Yamada et al. (2016), with an industrial instance named “Company_B”.

Table 4 provides information about the System Under Test of each instance, where $S_P$ is the number of parameters and their domain (e.g. the meaning of $2^{29}3^{1}$ in instance 7 is that the instance contains 29 parameters of domain 2 and 1 parameter of domain 3); $S_{\varphi }$ is the number of SUT constraints and their sizes (e.g. the meaning of $2^{13}3^{2}$ in instance 7 is that the instance contains 13 constraints of size 2 and 2 constraints of size 3); and *# lits* $CNF(S_{\varphi })$ is the number of literals of the CNF representation of $S_{\varphi }$ (i.e. the sum of the sizes of all clauses).

Table 4 also reports, for $t=2$, the following data: $ub^{ACTS}$, which indicates the upper bound returned by the ACTS tool (see Sect. 4); $ub^{\simeq }$, which is the best known upper bound (a star indicates that it is optimal, i.e., *CAN*(2, *S*)); *lb*, which reports the lower bound (computed as in Sect. 4); and $|\mathcal {T}_a|$ and $|\mathcal {T}_f|$, which report the number of allowed and forbidden tuples, respectively.

Finally, we also show, for the $PMSat_{CCX}^{N,t=2,S,lb}$ encoding of each instance, the following information: *# vars*, which is the number of variables used by this encoding; *# clauses*, which is the number of clauses; *# lits*, which is the number of literals; and *size (MB)*, which is the file size of the WCNF formula in MB.

Notice that in this paper we focus on $t=2$ strength coverage.

**Table 4:** General information of all benchmarks used

Instance	System Under Test (SUT)			Bounds for $t = 2$					$PMSat_{CCX}^{N,t=2,S,lb}$			
	$S_P$	$S_{\varphi }$	# lits $CNF(S_{\varphi })$	$ub^{ACTS}$	$ub^{\simeq }$	lb	$|\mathcal {T}_a|$	$|\mathcal {T}_f|$	# vars	# clauses	# lits	size (MB)
Cohen et al. (2008)												
1	$2^{86}3^{3}4^{1}5^{5}6^{2}$	$2^{20}3^{3}4^{1}$	53	48	37	35	23876	474	1158588	2620675	7463282	60.01
2	$2^{86}3^{3}4^{3}5^{1}6^{1}$	$2^{19}3^{3}$	47	32	30*	29	20331	237	657890	1371738	3984183	29.91
3	$2^{27}4^{2}$	$2^{9}3^{1}$	21	19	18*	15	1838	14	36217	79008	222390	1.47
4	$2^{51}3^{4}4^{2}5^{1}$	$2^{15}3^{2}$	36	22	20*	19	7530	386	168852	358291	1025536	7.33
5	$2^{155}3^{7}4^{3}5^{5}6^{4}$	$2^{32}3^{6}4^{1}$	86	54	45	35	76259	73	4142574	9720622	27451121	236.74
6	$2^{73}4^{3}6^{1}$	$2^{26}3^{4}$	64	25	24*	23	11382	1878	289001	597814	1730859	12.72
7	$2^{29}3^{1}$	$2^{13}3^{2}$	32	12	9	5	1567	231	19566	49758	132435	0.85
8	$2^{109}3^{2}4^{2}5^{3}6^{3}$	$2^{32}3^{4}4^{1}$	80	47	36*	35	33680	1098	1597165	3590459	10247230	84.50
9	$2^{57}3^{1}4^{1}5^{1}6^{1}$	$2^{30}3^{7}$	81	22	20*	19	6835	1720	153584	325984	932515	6.63
10	$2^{130}3^{6}4^{5}5^{2}6^{4}$	$2^{40}3^{7}$	101	47	41	35	52659	2029	2493173	5608369	16010703	135.34
11	$2^{84}3^{4}4^{2}5^{2}6^{4}$	$2^{28}3^{4}$	68	47	39	35	23636	707	1123311	2523897	7200149	57.70
12	$2^{136}3^{4}4^{3}5^{1}6^{3}$	$2^{23}3^{4}$	58	43	36*	35	49522	978	2144718	4675267	13461992	108.23
13	$2^{124}3^{4}4^{1}5^{2}6^{2}$	$2^{22}3^{4}$	56	40	36*	35	38862	1701	1567084	3319517	9632256	75.77
14	$2^{81}3^{5}4^{3}6^{3}$	$2^{13}3^{2}$	32	39	36*	35	20544	618	810618	1697204	4936072	37.18
15	$2^{50}3^{4}4^{1}5^{2}6^{1}$	$2^{20}3^{2}$	46	32	30*	29	8388	155	273410	569181	1650514	12.10
16	$2^{81}3^{3}4^{2}6^{1}$	$2^{30}3^{4}$	72	25	24*	23	14600	2303	370051	765960	2218422	16.44
17	$2^{128}3^{3}4^{2}5^{1}6^{3}$	$2^{25}3^{4}$	62	41	36*	35	43390	66	1792402	3835891	11100545	88.14
18	$2^{127}3^{2}4^{4}5^{6}6^{2}$	$2^{23}3^{4}4^{1}$	62	52	41	35	50128	28	2625808	6092947	17250882	146.38
19	$2^{172}3^{9}4^{9}5^{3}6^{4}$	$2^{38}3^{5}$	91	51	43	35	98778	114	5064366	11694341	33170488	287.31
20	$2^{138}3^{4}4^{5}5^{4}6^{7}$	$2^{42}3^{6}$	102	60	54	35	64620	3320	3903864	9411047	26386102	227.62
21	$2^{76}3^{3}4^{2}5^{1}6^{3}$	$2^{40}3^{6}$	98	39	36*	35	15442	2742	610938	1279170	3717471	27.90
22	$2^{72}3^{4}4^{1}6^{2}$	$2^{20}3^{2}$	46	37	36*	35	13405	1181	503127	1028139	3008516	22.48
23	$2^{25}3^{1}6^{1}$	$2^{13}3^{2}$	32	14	12*	11	1495	173	21856	47740	132915	0.85
24	$2^{110}3^{2}5^{3}6^{4}$	$2^{25}3^{4}$	62	48	41	35	34204	570	1656252	3748659	10679658	88.30
25	$2^{118}3^{6}4^{2}5^{2}6^{6}$	$2^{23}3^{3}4^{1}$	59	52	49	35	46968	52	2461280	5710735	16167454	136.81
26	$2^{87}3^{1}4^{3}5^{4}$	$2^{28}3^{4}$	68	34	26	24	20921	667	719347	1643461	4647485	36.52
27	$2^{55}3^{2}4^{2}5^{1}6^{2}$	$2^{17}3^{3}$	43	37	36*	35	9714	43	365524	746797	2183919	16.18
28	$2^{167}3^{16}4^{2}5^{3}6^{6}$	$2^{31}3^{6}$	80	57	50	35	96599	74	5535861	13181074	37087871	322.33
29	$2^{134}3^{7}5^{3}$	$2^{19}3^{3}$	47	29	25*	24	45839	32	1338905	2899941	8321499	64.34
30	$2^{73}3^{3}4^{3}$	$2^{31}3^{4}$	74	22	16*	15	12453	1308	277976	640938	1792681	13.16
apache	$2^{158}3^{8}4^{4}5^{1}6^{1}$	$2^{3}3^{1}4^{2}5^{1}$	22	33	30*	29	66927	3	2221926	4701044	13619419	109.16
bugzilla	$2^{49}3^{1}4^{2}$	$2^{4}3^{1}$	11	19	16*	15	5818	4	112768	247130	697953	4.82
gcc	$2^{189}3^{10}$	$2^{37}3^{3}$	83	23	15	8	82770	39	1913568	5063264	13685896	112.78
spins	$2^{13}4^{5}$	$2^{13}$	26	26	19*	15	979	13	27050	64498	177169	1.23
spinv	$2^{42}3^{2}4^{11}$	$2^{47}3^{2}$	100	45	33	15	8741	56	401069	1063265	2888090	22.53
Segall et al. (2011)												
Banking1	$3^{4}4^{1}$	$5^{112}$	560	15	13*	11	102	0	1938	5864	19573	0.11
Banking2	$2^{14}4^{1}$	$2^{3}$	6	11	10*	7	473	3	5591	12845	34672	0.21
CommProtocol	$2^{10}7^{1}$	$2^{10}3^{10}4^{12}5^{24}$ $6^{30}7^{30}8^{12}$	704	19	16*	13	285	35	6047	15914	50363	0.29
Concurrency	$2^{5}$	$2^{4}3^{1}5^{2}$	21	6	5*	3	36	4	278	667	1686	0.01
Healthcare1	$2^{6}3^{2}5^{1}6^{1}$	$2^{3}3^{18}$	60	30	30*	29	361	8	12090	24661	70619	0.44
Healthcare2	$2^{5}3^{6}4^{1}$	$2^{1}3^{6}5^{18}$	110	16	14	11	466	1	8212	18853	52268	0.31
Healthcare3	$2^{16}3^{6}4^{5}5^{1}6^{1}$	$2^{31}$	62	38	34*	29	3092	59	121950	271023	768538	5.38
Healthcare4	$2^{13}3^{12}4^{6}5^{2}6^{1}7^{1}$	$2^{22}$	44	49	46*	41	5707	38	287980	619634	1783211	13.14
Insurance	$2^{6}3^{1}5^{1}6^{2}11^{1}13^{1}17^{1}31^{1}$	–	0	527	527*	526	4573	0	2509047	5009492	14863678	122.95
NetworkMgmt	$2^{2}4^{1}5^{3}10^{2}11^{1}$	$2^{20}$	40	112	110*	109	1228	20	148402	301059	877206	6.28
ProcessorComm1	$2^{3}3^{6}4^{6}$	$2^{13}$	26	29	21	15	1058	13	32957	80475	219601	1.54
ProcessorComm2	$2^{3}3^{12}4^{8}5^{2}$	$1^{4}2^{121}$	246	32	25*	24	2525	854	85287	193248	541399	3.73
Services	$2^{3}3^{4}5^{2}8^{2}10^{2}$	$3^{386}4^{2}$	1166	106	100*	99	1819	16	204692	460866	1346965	9.78
Storage1	$2^{1}3^{1}4^{1}5^{1}$	$4^{95}$	380	17	17*	14	53	18	1294	4270	13468	0.07
Storage2	$3^{4}6^{1}$	–	0	18	18*	17	126	0	2826	5652	15552	0.09
Storage3	$2^{9}3^{1}5^{3}6^{1}8^{1}$	$2^{38}3^{10}$	106	50	50*	39	1020	120	54810	122009	344328	2.47
Storage4	$2^{5}3^{7}4^{1}5^{2}6^{2}7^{1}10^{1}13^{1}$	$2^{24}$	48	136	130*	129	3491	24	495046	1012862	2970799	22.45
Storage5	$2^{5}3^{8}5^{3}6^{2}8^{1}9^{1}10^{2}11^{1}$	$2^{151}$	302	218	215	109	5342	246	1206084	3020149	8366680	72.16
SystemMgmt	$2^{5}3^{4}5^{1}$	$2^{13}3^{4}$	38	17	15*	14	310	14	5935	12813	35376	0.21
Telecom	$2^{5}3^{1}4^{2}5^{1}6^{1}$	$2^{11}3^{1}4^{9}$	61	32	30*	29	440	11	15650	32761	93262	0.59
Yu et al. (2015)												
RL-A	$2^{5}3^{4}4^{7}5^{4}6^{5}$ $7^{4}8^{1}12^{3}$	$1^{12}2^{491}3^{345}$	2029	155	153	143	7066	7156	1142671	2491775	7220414	59.32
RL-B	$2^{8}3^{2}4^{3}5^{3}6^{1}9^{1}$ $10^{1}12^{2}$	$1^{8}2^{1127}3^{277}$	27721	767	727	519	17018	5597	13365222	35733711	109278283	1026.60
	$$14^{3}20^{1}24^{1}37^{1}$$	$$4^{1755}5^{1064}6^{2048} $$										
Yamada et al. (2016)												
Company2	$2^{6}3^{4}8^{4}$	$1^{2}2^{35}3^{89}4^{54}5^{34}$	1247	81	72	55	1149	261	100546	252543	744203	5.35
		$$6^{20}7^{34}8^{16}9^{4}$$										

Regarding existing tools for solving Mixed Covering Arrays with Constraints, the main tool we compare with is CALOT (Yamada et al. 2015). Unfortunately, CALOT is not available from the authors but we did our best to reproduce it (see Sect. 6), showing our experimental investigation that the results are consistent with those of Yamada et al. (2015). Our implementation of CALOT and all algorithms presented in this paper can be found in http://hardlog.udl.cat/static/doc/inc-maxsat-ct/html/index.html, which we think is also a nice contribution for both the combinatorial testing and satisfiability communities.

Since all the algorithms presented in this paper are built on top of a SAT solver, we compared, when possible, all the algorithms with the same underlying SAT solver. That is not the case in Yamada et al. (2015), which may lead to flawed conclusions. In our experimental investigation we choose Glucose (version 4.1) (Audemard et al. 2013), as most of the state-of-the-art MaxSAT solvers are built on top of it.

We also use the ACTS tool (Borazjany et al. 2012) to compute fast and good enough upper bounds of the Covering Array Number problem, although it is not competitive with SAT-based approaches.

The environment of execution consists of a computer cluster with machines equipped with two Intel Xeon Silver 4110 (octa-core processors at 2.1GHz, 11MB cache memory) and 96GB DDR4 main memory. Unless otherwise stated, all the experiments were executed with a timeout of 2h and a memory limit of 18GB. To mitigate the impact of randomness we executed all the algorithms using five different seeds for each instance.

The rest of the experimental section is organized as follows. Regarding the Covering Array Number, in Sect. 11.1, we compare the CALOT algorithm with the MaxSAT encodings and SAT-based MaxSAT approaches described in Sects. 7 and 8 . Regarding the Tuple Number problem, in Sect. 11.2, we evaluate the complete and incomplete MaxSAT algorithms on the encoding described in Sect. 9. Then, in Sect. 11.3, we evaluate the incomplete approach for computing the Tuple Number described in Sect. 10.

### SAT-based MaxSAT approaches for the covering array number problem

In this experiment, we compare the performance of state-of-the-art SAT-based MaxSAT solvers with the CALOT algorithm described in Sect. 6. We hypothesise that since these SAT-based MaxSAT algorithms, once executed on the suitable MaxSAT encodings, can *simulate* the behaviour of the CALOT algorithm (see Propositions 6 and 8) but the opposite is not true, MaxSAT algorithms may perform similarly or outperform the CALOT algorithm. This hypothesis would contradict the findings in Yamada et al. (2015), where it was reported that the CALOT algorithm clearly dominates the MaxSAT-based approach in Ansótegui et al. (2013). If our hypothesis is correct, MaxSAT approaches for solving the Covering Array Number problem would be put back on the agenda. We focus in anytime algorithms that must be able to report suboptimal solutions.^10^

**Solvers** The CALOT algorithm (described in Sect. 6) and the model-guided Linear SAT-based MaxSAT algorithm Linear (described in Sect. 8) were implemented on top of the OptiLog (Ansótegui et al. 2021) python framework for SAT solving. This framework includes python bindings for several state-of-the-art SAT solvers and the python binding to the PBLib (Logic and Optimization Group 2019).

We additionally tested several complete and incomplete algorithms from the MaxSAT Evaluation 2020 (Bacchus et al. 2020). From complete MaxSAT solvers we tested MaxHS (Bacchus 2020), EvalMaxSAT (Avellaneda 2020), RC2 (Ignatiev 2020) and maxino (Alviano et al. 2015). We only report results for RC2 and one seed,^11^ as this was the complete solver that reported better results. MaxHS obtained the best results for 2 of the tested instances, but we decided to exclude it from the comparison since it cannot report upper bounds for most of the instances and it uses another underlying SAT solver than Glucose41.

Regarding incomplete MaxSAT algorithms we tested Berg et al. (2020), tt-open-wbo-inc (Nadel 2020) and SatLike (Lei and Cai 2018). We report results for Loandra and tt-open-wbo-inc as SatLike crashed in some of the tested instances.

**MaxSAT encodings** We report results on $PMSat_{CCX}^{N,t,S,lb}$ and the weighted version $WPMSat_{CCX}^{N,t,S,lb}$ using a linear increase for the weights ($w_i = i-(lb+2)+1$, see Eq. *WSoftU* in Sect. 7). We found that $WPMSat_{CCX}^{N,t,S,lb}$ with the linear and exponential increase ($w_i = 2^{i-(lb+2)}$) lead to the same performance, but the exponential increase represented a problem for some MaxSAT solvers when *i* was high enough.

We further tested the three different alternatives for equation (a) from *CCX*, where two reported good results. The first one is the original (a) equation shown in Sect. 3, $(c^{i}_{\tau } \rightarrow c^{i-1}_{\tau } \vee x_{i,p,v})$, which we will refer to as a.0. The second one is the variation $(c^{i}_{\tau } \rightarrow c^{i-1}_{\tau } \vee x_{i,p,v}) \wedge (c^{i}_{\tau } \leftarrow c^{i-1}_{\tau })$, which we will refer to as a.1.

**Results** Table 5 shows the results of our experimentation. For each row and solver column, we give the average size of the minimum MCAC (out of the 5 executions per instance) and the average runtime. Bold values represent the best results. In case there are ties in size, the best time is marked. Sizes that have a star represent that the optimum has been certified in at least one of the five seeds executed for the current benchmark instance.

Table 6 aggregates the information presented in Table 5 to analyze the dominance relations among approaches. In particular, we show for each row the number of wins (W) and loses (L) with respect to each of the approaches in columns, for both size and run times. We consider that if algorithm A finds a smaller MCAC than B, then A also needs less runtime than B. In this sense, we will say that an approach outperforms another if it provides a strictly better solution within the given timeout or finds the same best suboptimal solution faster. For example, in the *ACTS* row we found that it obtains worse sizes than CALOT CCX a.0 in 52 instances (0 W, 52 L in column *size*), better runtimes in 2 and worse runtimes in 56 (2 W, 56 L in column *time*).

**Table 5:** Comparison of SAT-based MaxSAT approaches versus CALOT for the Covering Array Number problem

Instance	ACTS		CALOT CCX a.0		CALOT CCX a.1		RC2-B CCX a.0		RC2-B CCX a.0 wpm		linear CCX a.0	
	Size	Time	Size	Time	Size	Time	Size	Time	Size	Time	Size	Time
Cohen et al. (2008)												
1	48.00	0.83	38.00	1688.39	38.00	1470.88	$-$1.00	$-$1.00	37.00	7146.52	37.00	5367.02
2	32.00	1.08	30.00*	2.80	30.00*	3.57	30.00*	9.85	30.00*	10.81	30.00*	2.91
3	19.00	1.21	18.00*	0.09	18.00*	0.12	18.00*	0.59	18.00*	0.73	18.00*	0.13
4	22.00	1.04	20.00*	0.52	20.00*	0.95	20.00*	2.58	20.00*	3.19	20.00*	0.74
5	54.00	1.31	47.00	1955.92	48.00	241.15	$-$1.00	$-$1.00	48.00	378.46	47.00	1693.29
6	25.00	1.11	24.00*	1.18	24.00*	1.46	24.00*	4.46	24.00*	5.47	24.00*	1.48
7	12.00	1.13	9.00	0.06	9.00	0.08	$-$1.00	$-$1.00	9.00	0.61	9.00	0.13
8	47.00	1.01	38.00	1403.51	37.00	4727.41	$-$1.00	$-$1.00	37.00	3407.87	38.00	547.77
9	22.00	1.21	20.00*	0.57	20.00*	0.84	20.00*	2.36	20.00*	2.66	20.00*	0.49
10	47.00	1.28	44.00	6860.63	44.20	5250.31	$-$1.00	$-$1.00	44.00	4759.95	44.00	1343.51
11	47.00	1.26	41.00	2641.09	41.00	4167.69	$-$1.00	$-$1.00	42.00	6172.69	42.00	1916.85
12	43.00	1.29	36.00*	14.13	36.00*	24.01	36.00*	36.81	36.00*	49.94	36.00*	20.66
13	40.00	1.15	36.00*	6.78	36.00*	9.80	36.00*	24.42	36.00*	29.91	36.00*	6.73
14	39.00	0.83	36.00*	3.76	36.00*	5.25	36.00*	13.81	36.00*	15.56	36.00*	4.80
15	32.00	1.05	30.00*	1.02	30.00*	1.49	30.00*	4.73	30.00*	4.88	30.00*	1.30
16	25.00	0.84	24.00*	1.36	24.00*	1.94	24.00*	5.59	24.00*	6.60	24.00*	1.65
17	41.00	1.17	36.00*	10.16	36.00*	13.60	36.00*	30.57	36.00*	35.75	36.00*	10.98
18	52.00	1.30	42.00	128.65	42.00	201.42	$-$1.00	$-$1.00	42.00	177.28	42.00	202.10
19	51.00	1.43	46.00	178.56	45.00	118.23	$-$1.00	$-$1.00	46.00	265.76	46.00	128.89
20	60.00	1.53	57.00	129.90	57.00	224.31	$-$1.00	$-$1.00	57.00	236.47	56.00	1092.85
21	39.00	1.33	36.00*	2.59	36.00*	3.86	36.00*	9.99	36.00*	9.95	36.00*	3.40
22	37.00	1.08	36.00*	1.75	36.00*	2.70	36.00*	7.74	36.00*	8.57	36.00*	2.17
23	14.00	0.75	12.00*	0.05	12.00*	0.09	12.00*	0.42	12.00*	0.41	12.00*	0.08
24	48.00	1.40	44.40	4172.55	45.00	185.06	$-$1.00	$-$1.00	44.00	41.40	45.00	13.58
25	52.00	1.28	52.00	100.56	52.00	117.86	$-$1.00	$-$1.00	$-$1.00	$-$1.00	52.00	423.91
26	34.00	1.37	27.00	49.99	27.00	31.54	$-$1.00	$-$1.00	27.00	112.13	27.00	551.57
27	37.00	1.32	36.00*	1.14	36.00*	1.68	36.00*	5.35	36.00*	6.70	36.00*	1.33
28	57.00	1.54	53.00	95.96	53.00	78.94	$-$1.00	$-$1.00	53.00	330.79	53.00	120.36
29	29.00	1.15	25.00*	5.63	25.00*	8.77	25.00*	20.73	25.00*	26.38	25.00*	7.81
30	22.00	0.81	16.00*	1.38	16.00*	1.75	16.00*	4.53	16.00*	5.98	16.00*	1.56
apache	33.00	1.25	30.00*	8.96	30.00*	13.68	30.00*	33.44	30.00*	38.18	30.00*	11.28
bugzilla	19.00	1.03	16.00*	0.35	16.00*	0.61	16.00*	1.72	16.00*	1.79	16.00*	0.36
gcc	23.00	0.93	15.00	13.09	15.00	19.06	$-$1.00	$-$1.00	15.00	47.85	15.00	22.14
spins	26.00	1.22	19.00*	**0.13**	19.00*	0.22	19.00*	0.56	19.00*	0.70	19.00*	0.16
spinv	45.00	0.84	33.00	5.28	32.00	188.97	$-$1.00	$-$1.00	32.00	55.75	33.00	21.39
Segall et al. (2011)												
Banking1	15.00	0.85	13.00*	**0.01**	13.00*	0.01	13.00*	0.20	13.00*	0.29	13.00*	0.08
Banking2	11.00	0.46	10.00*	**0.01**	10.00*	0.02	10.00*	25.56	10.00*	22.95	10.00*	0.03
CommProtocol	19.00	1.05	16.00*	**0.02**	16.00*	0.03	16.00*	0.19	16.00*	0.22	16.00*	0.03
Concurrency	6.00	0.49	5.00*	**0.00**	5.00*	0.00	5.00*	0.08	5.00*	0.08	5.00*	0.01
Healthcare1	30.00	0.55	30.00*	0.03	30.00*	0.03	30.00*	0.24	30.00*	0.25	30.00*	0.05
Healthcare2	16.00	0.60	**14.00**	**0.02**	14.00	0.07	$-$1.00	$-$1.00	14.00	0.21	14.00	0.07
Healthcare3	38.00	0.58	34.00*	0.48	34.00*	0.75	34.00*	2.14	34.00*	2.39	34.00*	0.68
Healthcare4	49.00	0.59	46.00*	1.77	46.00*	2.33	46.00*	5.88	46.00*	7.24	46.00*	2.61
Insurance	**527.00**	**0.31**	527.00*	16.82	527.00*	18.56	527.00*	44.42	527.00*	45.47	527.00*	21.08
NetworkMgmt	112.00	0.58	110.00*	214.18	110.00*	511.52	110.00*	180.82	110.00*	233.46	110.00*	1100.79
ProcessorComm1	29.00	0.52	21.00	67.53	22.00	0.47	$-$1.00	$-$1.00	22.00	1.14	**21.00**	**28.65**
ProcessorComm2	32.00	0.77	25.00*	0.42	25.00*	0.55	25.00*	1.42	25.00*	1.70	25.00*	0.42
Services	106.00	1.20	100.00*	28.65	100.00*	29.89	100.00*	40.55	100.00*	37.37	100.00*	26.69
Storage1	17.00	0.75	17.00*	**0.00**	17.00*	0.01	17.00*	0.11	17.00*	0.11	17.00*	0.01
Storage2	18.00	0.25	18.00*	**0.01**	18.00*	0.01	18.00*	0.12	18.00*	0.12	18.00*	0.02
Storage3	50.00	0.66	50.00*	0.14	50.00*	0.28	50.00*	11.02	50.00*	1.86	50.00*	0.26
Storage4	136.00	0.60	130.00*	2.49	130.00*	3.24	130.00*	7.24	130.00*	9.28	130.00*	1.69
Storage5	218.00	0.87	215.00	68.48	215.00	389.33	$-$1.00	$-$1.00	215.00	140.68	215.00	811.43
SystemMgmt	17.00	0.57	15.00*	**0.01**	15.00*	0.03	15.00*	0.16	15.00*	0.18	15.00*	0.02
Telecom	32.00	0.58	30.00*	0.04	30.00*	0.05	30.00*	0.31	30.00*	0.37	30.00*	0.06
Yu et al. (2015)												
RL-A	155.00	2.88	153.00	14.02	153.00	25.95	$-$1.00	$-$1.00	153.00	28.52	153.00	18.89
RL-B	767.00	170.61	760.00	4471.65	763.00	5862.61	$-$1.00	$-$1.00	$-$1.00	$-$1.00	764.00	5633.81

Instance	loandra CCX a.0		loandra CCX a.1		tt-open-wbo-inc CCX a.0		tt-open-wbo-inc CCX a.1		tt-open-wbo-inc CCX a.0 wpm		tt-open-wbo-inc CCX a.1 wpm	
	Size	Time	Size	Time	Size	Time	Size	Time	Size	Time	Size	Time
Yamada et al. (2016)												
Company2	81.00	9.37	72.00	1.80	72.00	1.88	$-$1.00	$-$1.00	72.00	3.33	72.00	2.48
Cohen et al. (2008)												
1	37.00	1718.97	**37.00**	**426.06**	37.00	902.46	38.00	133.26	37.00	4724.61	38.00	1196.04
2	30.00*	7.19	30.00*	7.68	**30.00**	**0.81**	30.00	1.42	30.00	0.92	30.00	1.70
3	18.00*	0.36	18.00*	0.47	18.00	0.06	18.00	0.08	**18.00**	**0.05**	18.00	0.07
4	20.00*	1.34	20.00*	2.24	20.00	0.30	**20.00**	**0.29**	20.00	0.31	20.00	0.45
5	46.00	1629.29	**45.80**	**1795.81**	46.00	1624.87	46.00	1348.31	46.20	1803.99	46.00	4655.60
6	24.00*	2.98	24.00*	3.51	**24.00**	**0.33**	24.00	0.48	24.00	0.34	24.00	0.47
7	9.00	30.98	9.00	30.57	9.00	0.06	9.00	0.06	**9.00**	**0.05**	9.00	0.06
8	36.80*	2849.90	37.00	3731.63	37.00	1178.58	**36.60**	**2948.62**	37.00	2333.83	37.00	5133.82
9	20.00*	1.22	20.00*	2.30	**20.00**	**0.18**	20.00	0.27	20.00	0.19	20.00	0.41
10	41.80	1235.36	41.00	1891.73	41.00	2720.18	41.00	970.10	41.00	5823.09	**41.00**	**641.10**
11	39.00	6308.22	40.00	4323.47	40.00	741.96	40.00	1086.24	**39.00**	**1628.02**	39.00	2892.01
12	36.00*	36.16	36.00*	42.45	**36.00**	**8.18**	36.00	10.46	36.00	10.75	36.00	11.37
13	36.00*	14.91	36.00*	25.04	36.00	3.46	36.00	3.31	**36.00**	**3.23**	36.00	3.60
14	36.00*	8.84	36.00*	11.51	36.00	1.89	36.00	2.48	**36.00**	**1.51**	36.00	4.28
15	30.00*	2.91	30.00*	3.70	**30.00**	**0.47**	30.00	0.66	30.00	0.90	30.00	0.77
16	24.00*	4.30	24.00*	4.46	**24.00**	**0.56**	24.00	0.60	24.00	0.57	24.00	0.62
17	36.00*	22.72	36.00*	31.18	**36.00**	**5.88**	36.00	7.48	36.00	7.31	36.00	6.94
18	41.00	637.62	41.00	734.99	**41.00**	**134.63**	41.00	626.83	41.00	2943.99	41.00	1871.62
19	44.00	2056.11	**43.80**	**2638.11**	44.00	755.22	44.00	756.79	44.00	2892.85	44.00	578.78
20	54.80	1422.09	**54.60**	**1736.92**	55.00	32.47	55.00	500.42	55.00	437.94	55.00	176.33
21	36.00*	7.08	36.00*	8.88	**36.00**	**1.06**	36.00	1.38	36.00	1.72	36.00	1.94
22	36.00*	5.25	36.00*	6.78	**36.00**	**0.65**	36.00	0.88	36.00	0.72	36.00	1.04
23	12.00*	0.22	12.00*	0.30	**12.00**	**0.03**	12.00	0.05	12.00	0.06	12.00	0.09
24	42.00	1693.19	**41.00**	**4304.36**	42.00	1465.17	43.00	59.40	42.00	884.17	42.00	926.51
25	50.00	2069.85	**49.80**	**1133.70**	51.00	136.11	50.00	2101.71	50.00	5892.75	50.00	263.99
26	27.00	103.46	27.00	57.66	26.00	6740.38	**26.00**	**790.74**	27.00	210.04	27.00	141.21
27	36.00*	2.96	36.00*	3.56	**36.00**	**0.39**	36.00	0.61	36.00	0.45	36.00	0.62
28	51.20	333.93	51.00	1236.07	51.00	3112.51	51.00	607.78	52.00	61.99	**49.00**	**3329.73**
29	25.00*	12.69	25.00*	17.48	**25.00**	**3.05**	25.00	4.02	25.00	3.55	25.00	4.63
30	16.00*	3.57	16.00*	3.94	**16.00**	**0.68**	16.00	0.83	16.00	0.77	16.00	1.08
apache	30.00*	21.65	30.00*	32.14	**30.00**	**5.17**	30.00	6.70	30.00	5.82	30.00	5.57
bugzilla	16.00*	0.89	16.00*	1.19	**16.00**	**0.14**	16.00	0.19	16.00	0.14	16.00	0.46
gcc	15.00	63.99	15.00	68.18	**15.00**	**9.44**	15.00	12.76	15.00	11.82	15.00	29.62
spins	19.00*	0.28	19.00*	0.28	19.00	0.13	19.00	0.21	19.00	0.20	19.00	0.21
spinv	32.00	61.37	32.00	97.82	**32.00**	**26.18**	32.00	82.41	32.00	53.36	32.00	199.38
Segall et al. (2011)												
Banking1	13.00*	0.05	13.00*	0.05	13.00	0.09	13.00	0.08	13.00	0.06	13.00	0.06
Banking2	10.00*	19.30	10.00*	10.62	10.00	0.02	10.00	0.02	10.00	0.02	10.00	0.02
CommProtocol	16.00*	0.15	16.00*	0.09	16.00	0.05	16.00	0.03	16.00	0.02	16.00	0.02
Concurrency	5.00*	0.03	5.00*	0.03	5.00	0.03	5.00	0.01	5.00	0.01	5.00	0.01
Healthcare1	30.00*	0.13	30.00*	0.15	30.00	0.17	30.00	0.03	**30.00**	**0.02**	30.00	0.03
Healthcare2	14.00	30.55	14.00	30.06	14.00	0.21	14.00	0.33	14.00	0.05	14.00	0.35
Healthcare3	34.00*	1.63	34.00*	1.44	**34.00**	**0.26**	34.00	0.32	34.00	0.37	34.00	0.45
Healthcare4	46.00*	3.64	46.00*	3.71	**46.00**	**0.73**	46.00	0.82	46.00	1.83	46.00	2.22
Insurance	527.00*	45.23	527.00*	46.40	527.00	5.29	527.00	10.47	527.00	5.08	527.00	11.25
NetworkMgmt	110.00*	45.65	110.00*	**34.19**	110.00	344.68	110.00	302.44	110.00	408.88	110.00	276.77
ProcessorComm1	21.00	468.17	21.00	107.68	21.00	610.20	21.00	1690.90	22.00	0.22	21.00	1015.43
ProcessorComm2	25.00*	0.78	25.00*	1.22	**25.00**	**0.23**	25.00	0.39	25.00	0.26	25.00	0.41
Services	100.00*	3.94	100.00*	3.97	100.00	2.43	100.00	2.44	100.00	2.42	**100.00**	**1.46**
Storage1	$-$1.00	$-$1.00	$-$1.00	$-$1.00	17.00	0.01	17.00	0.01	17.00	0.01	17.00	0.01
Storage2	18.00*	0.05	18.00*	0.05	18.00	0.02	18.00	0.01	18.00	0.01	18.00	0.01
Storage3	$-$1.00	$-$1.00	$-$1.00	$-$1.00	50.00	0.09	50.00	0.11	**50.00**	**0.08**	50.00	0.12
Storage4	130.00*	5.16	130.00*	6.65	**130.00**	**0.55**	130.00	0.84	130.00	0.58	130.00	0.83
Storage5	215.00	85.07	215.00	90.98	215.00	17.01	**215.00**	**16.83**	215.00	45.27	215.00	46.54
SystemMgmt	15.00*	0.08	15.00*	0.09	15.00	0.02	15.00	0.02	15.00	0.02	15.00	0.02
Telecom	30.00*	0.18	30.00*	0.22	30.00	0.03	30.00	0.04	**30.00**	**0.03**	30.00	0.04
Yu et al. (2015)												
RL-A	153.00	58.49	153.00	63.11	**153.00**	**3.25**	153.00	3.77	153.00	12.04	153.00	15.40
RL-B	$-$1.00	$-$1.00	$-$1.00	$-$1.00	$-$1.00	$-$1.00	**727.00**	**966.65**	$-$1.00	$-$1.00	727.00	5809.65
Yamada et al. (2016)												
Company2	72.00	32.15	72.00	32.96	**72.00**	**0.68**	72.00	0.75	72.00	0.99	72.00	1.29

Bold values represent the best results. In case of ties in size, the best time is marked. For sizes with a star the optimum has been certified in at least one of the five seeds executed

**Table 6:** Dominance relations for CALOT and SAT-based MaxSAT approaches for the Covering Array Number problem

	CALOT CCX a.0		CALOT CCX a.1		RC2-B CCX a.0		RC2-B CCX a.0 wpm		linear CCX a.0		loandra CCX a.0	
	Size	Time	Size	Time	Size	Time	Size	Time	Size	Time	Size	Time
	W L	W L	W L	W L	W L	W L	W L	W L	W L	W L	W L	W L
ACTS	0 **52**	2 **56**	0 **52**	2 **56**	21 **32**	23 **35**	2 **51**	4 **54**	0 **52**	2 **56**	3 **52**	4 **54**
CALOT CCX a.0	–	–	**5** 3	**52** 6	**21** 0	**57** 1	**5** 4	**53** 5	**3** 2	**47** 11	3 **12**	**44** 14
CALOT CCX a.1	–	–	–	–	**21** 0	**57** 1	**4** 3	**50** 8	5 **5**	27 **31**	3 **12**	**42** 16
RC2-B CCX a.0	–	–	–	–	–	–	0 **19**	**31** 25	0 **21**	1 **57**	2 **20**	3 **54**
RC2-B CCX a.0 wpm	–	–	–	–	–	–	–	–	3 **5**	7 **51**	2 **11**	8 **49**
linear CCX a.0	–	–	–	–	–	–	–	–	–	–	3 **11**	**41** 17
loandra CCX a.0	–	–	–	–	–	–	–	–	–	–	–	–
loandra CCX a.1	–	–	–	–	–	–	–	–	–	–	–	–
tt-open-wbo-inc CCX a.0	–	–	–	–	–	–	–	–	–	–	–	–
tt-open-wbo-inc CCX a.1	–	–	–	–	–	–	–	–	–	–	–	–
tt-open-wbo-inc CCX a.0 wpm	–	–	–	–	–	–	–	–	–	–	–	–

	loandra CCX a.1		tt-open-wbo-inc CCX a.0		tt-open-wbo-inc CCX a.1		tt-open-wbo-inc CCX a.0 wpm		tt-open-wbo-inc CCX a.1 wpm			
	size	time	size	time	size	time	size	time	size	time		
	W L	W L	W L	W L	W L	W L	W L	W L	W L	W L		
ACTS	3 **52**	4 **54**	1 **52**	2 **56**	0 **53**	1 **57**	1 **52**	2 **56**	0 **53**	1 **57**		
CALOT CCX a.0	3 **12**	**44** 14	1 **13**	13 **45**	0 **13**	12 **46**	2 **12**	15 **43**	0 **12**	19 **39**		
CALOT CCX a.1	3 **11**	**42** 16	1 **12**	9 **49**	0 **13**	6 **52**	1 **10**	7 **51**	0 **11**	11 **47**		
RC2-B CCX a.0	2 **20**	6 **51**	0 **20**	1 **56**	0 **21**	1 **57**	0 **20**	1 **56**	0 **21**	1 **57**		
RC2-B CCX a.0 wpm	2 **10**	10 **47**	0 **11**	1 **56**	1 **13**	4 **54**	0 **9**	2 **55**	1 **11**	6 **52**		
linear CCX a.0	3 **11**	**41** 17	1 **12**	7 **51**	1 **13**	4 **54**	2 **11**	3 **55**	1 **12**	7 **51**		
loandra CCX a.0	2 **7**	**38** 17	4 **5**	9 **48**	4 **7**	9 **49**	**5** 3	12 **45**	3 **5**	10 **48**		
loandra CCX a.1	–	–	**5** 3	13 **44**	**6** 5	9 **49**	**7** 3	13 **44**	**6** 5	13 **45**		
tt-open-wbo-inc CCX a.0	–	–	–	–	2 **3**	**39** 19	**4** 2	**38** 19	2 **4**	**42** 16		
tt-open-wbo-inc CCX a.1	–	–	–	–	–	–	**6** 3	21 **37**	2 **3**	**39** 19		
tt-open-wbo-inc CCX a.0 wpm	–	–	–	–	–	–	–	–	1 **4**	**43** 15		

Bold values highlight winning algorithm per size or runtime

We observe how both *tt-open-wbo-inc* and *loandra* outperform the results obtained by *CALOT*, improving the sizes in more than 10 of the 58 available instances and, in the case of *tt-open-wbo-inc*, we also improve runtimes in more than 40 instances. This confirms our hypothesis that MaxSAT approaches can *simulate* and even improve the results obtained by the *CALOT* algorithm.

Regarding the different variations of the *CCX* encoding, we notice that for *tt-open-wbo-inc* and *loandra*, variation a.1 slightly improves results obtained by the original variation a.0. In particular, we observe that *tt-open-wbo-inc* with this specific encoding obtains the best size^12^ in instance *RL-B* (727), while algorithm *CALOT* reports a size of 760. However, this behaviour of the encoding a.1 is not observed in algorithm *CALOT*, as in this case, the best variation of equation (a) seems to be a.0. These results suggest that in case we use a new MaxSAT solver we should not discard at front any encoding variation.

For *RC2* and *linear* approaches we can observe clear differences among them when applying the $PMSat_{CCX}^{N,t,S,lb}$ encoding, as *linear* obtains better sizes and times in 21 and 57 instances respectively. These results show that, for the Covering Array Number problem, it is more effective to perform a search that incrementally refines the upper bound as the linear approach does (see Sect. 8). However, we observe a substantial improvement when using the $WPMSat_{CCX}^{N,t,S,lb}$ with the *RC2* MaxSAT solver, improving the sizes obtained by its unweighted counterpart in 19 of the 58 instances, which produces similar results than *CALOT* and $PMSat_{CCX}^{N,t,S,lb}$ *linear* approaches. This is expected since the weighted version forces RC2 to perform a top-down search as discussed in Sect. 8.

We also tested the $WPMSat_{CCX}^{N,t,S,lb}$ encoding over the *tt-open-wbo-inc*, a not core-guided MaxSAT solver. We observe that results are similar or slightly worse than with the $PMSat_{CCX}^{N,t,S,lb}$. We believe the $WPMSat_{CCX}^{N,t,S,lb}$ encoding is more useful for core-guided MaxSAT solvers as it modifies their refinement strategy (i.e. improve the upper bound instead of the lower bound). We also observed that refining the lower bound for the Covering Array Number problem is more challenging than refining the upper bound, as there are some instances where encoding $PMSat_{CCX}^{N,t,S,lb}$ with *RC2* (which would refine the lower bound) is not able to report any results, usually on instances where the CAN is not found.

### Weighted partial MaxSAT approaches for the tuple number problem

Encouraged by the good results of the proposed MaxSAT approaches for the Covering Array Number problem, we now evaluate the MaxSAT approach described in Sect. 9 on SAT-based MaxSAT approaches for solving the Tuple Number problem. Notice that the CALOT algorithm only works for solving the Covering Array Number problem. In this sense, this is a pioneering work on applying SAT technology to solve the Tuple Number problem.

**Solvers** We choose the *tt-open-wbo-inc* MaxSAT solver to perform these experiments, as this has been the approach that achieved better results in Sect. 11.1.

**MaxSAT encodings** We recall there are also some variations of the $TPMSat_{CCX}^{N,t,S,lb}$ encoding, due to the way constraint *CCX* is formulated, i.e. the relation among $c^{i}_{\tau }$ vars and $x_{i,p,v}$ vars (see Remark 1 in Sect. 3). According to some preliminary experimentation we observed that variation $(c^{i}_{\tau } \leftrightarrow c^{i-1}_{\tau } \vee x_{i,p,v})$, to which we refer as a.2, reported also good results, while variation a.1 did not and was excluded.

We additionally noticed that, when computing the tuple number, the cost of the solution returned by the MaxSAT solver when using the original encoding of equation (a) in *CCX*, $(c^{i}_{\tau } \rightarrow c^{i-1}_{\tau } \vee x_{i,p,v})$, can indeed overestimate the real cost of the solution induced by the value of the $x_{i,p,v}$ vars, i.e., the assignments that represent the actual tests used in the solution. This can happen since it is possible to set to False a $c^{i}_{\tau }$ even if the right-hand side of the implication is True. Enforcing the other side of the implication corrects this issue. For these reasons we will use the $(c^{i}_{\tau } \leftrightarrow c^{i-1}_{\tau } \vee x_{i,p,v})$ variation of *CCX*.

**Results** We would like to study the evolution of the number of covered tuples as a function of the number of tests, as we hypothesise that adding a new test close to the Covering Array Number (that guarantees all tuples can be covered) will allow adding very few additional tuples. In that sense, if these tests are expensive enough, they will not pay off in terms of the available budget and the additional percentage of coverage we can achieve.

**Fig. 1:**
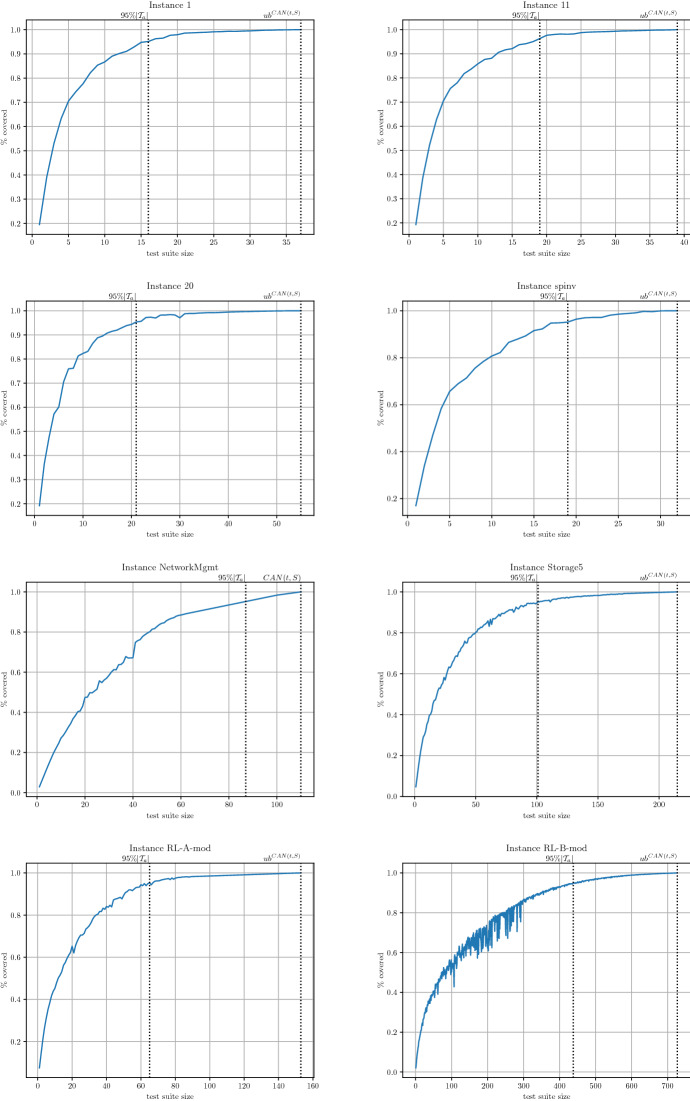
Number of tests required to reach a certain coverage percentage for the *tt-open-wbo-inc* approach

In Fig. 1, we show the number of tests required to reach a certain percentage of the tuples to cover for the *tt-open-wbo-inc* approach. Notice that *tt-open-wbo-inc* is an incomplete MaxSAT solver and we are therefore reporting a lower bound on the possible percentage by a particular number of tests. For lack of space, we only show the most representative instances of all the benchmark families.

We observe, for all the tested instances, that most of the tuples are covered using a relatively small number of tests and the remaining tuples require a relatively large additional number of tests. In our experiments, with only 52% of tests required for the Covering Array Number or for the best suboptimal solution from Table 5 in Sect. 11.1, we are able to reach a 95% coverage, whereas the remaining 5% of tuples need the remaining 48% of tests.

We also notice that the Tuple Number problem is more challenging than the Covering Array Number problem. According to some experimentation that we performed using complete MaxSAT solvers, none of the tested approaches has been able to certify any optimum for $N > 1$, even for the instances that were easy to solve for the Covering Array Number problem.

Another interesting observation is the erratic behavior on the *RL-B* instance (Yu et al. 2015) (Fig. 1, bottom right). *RL-B* is the biggest instance in the available benchmarks, having 27 parameters with domains up to 37, and with a suboptimal solution for the Covering Array Number (for $t=2$) of 727 tests. After 100 tests, the results for the Tuple Number problem become quite unstable in contrast to the behaviour on the rest of the instances. This phenomenon points out that the approach analyzed in this section has some limitations when instances are large enough. For a fixed set of parameters, instances become bigger when we increase the strength *t* or the number of tests as in this case.

To conclude this section, we have confirmed that MaxSAT is a good approach to solve the Tuple Number problem with constraints. We have also observed that with a relatively small number of tests we can cover most of the tuples, and that this approach can be useful for medium-sized instances that do not need a large number of tests to reach a reasonable coverage percentage.

In the next section, we explore the Incremental Test Suite Construction for the Tuple Number problem described in Sect. 10.1. It allows us to tackle more efficiently those Tuple Number problems involving a relatively large number of tests.

### MaxSAT based incremental test suite construction for *T*(*N*; *t*, *S*)

In Sect. 11.2, we have analyzed an approach that can be used to maximise the number of tuples covered by a number of tests inferior to *CAN*(*t*, *S*). However, we have seen that it becomes less efficient if we require to compute the Tuple Number problem for a large enough number of tests.

**Solving approaches** Here we propose three incomplete alternatives for solving the Tuple Number problem, with the aim of improving the results obtained in Sect. 11.2. Our hypothesis is that the application of incomplete approaches can be more suitable when solving bigger instances.

The first approach is the greedy algorithm presented in Yamada et al. (2016), referred to as $maxh-its$. This algorithm incrementally adds a test at a time. The test is constructed through a heuristic (Czerwonka 2006) that tries to increase the number of covered tuples so far, by selecting at each step the parameter tuple with the most value tuples yet to be covered.

The second approach is the Incremental Test Suite Construction from Sect. 10.1 (referred here as $maxsat-its$), which also adds a test at a time,^13^ but this test is built by solving the Tuple Number problem through an incomplete MaxSAT solver instead of using a heuristic as in the previous approach.

In the third approach, instead of a MaxSAT query, as in the second approach, we apply a SAT query to return a test that covers at least one more tuple (referred to as $sat-its$) than the incremental test suite built so far.

We also evaluate the approach described in Sect. 9.2. The idea is to relax the Covering Array Number problem by allowing to cover only a 95% of the allowed tuples ($\tau _a$). We refer to this approach as $mints-95\%|\tau _a|$. As for the Covering Array Number problem, we use the upper bound returned by the ACTS tool (see Sect. 4) for the initial number of tests.

**Fig. 2:**
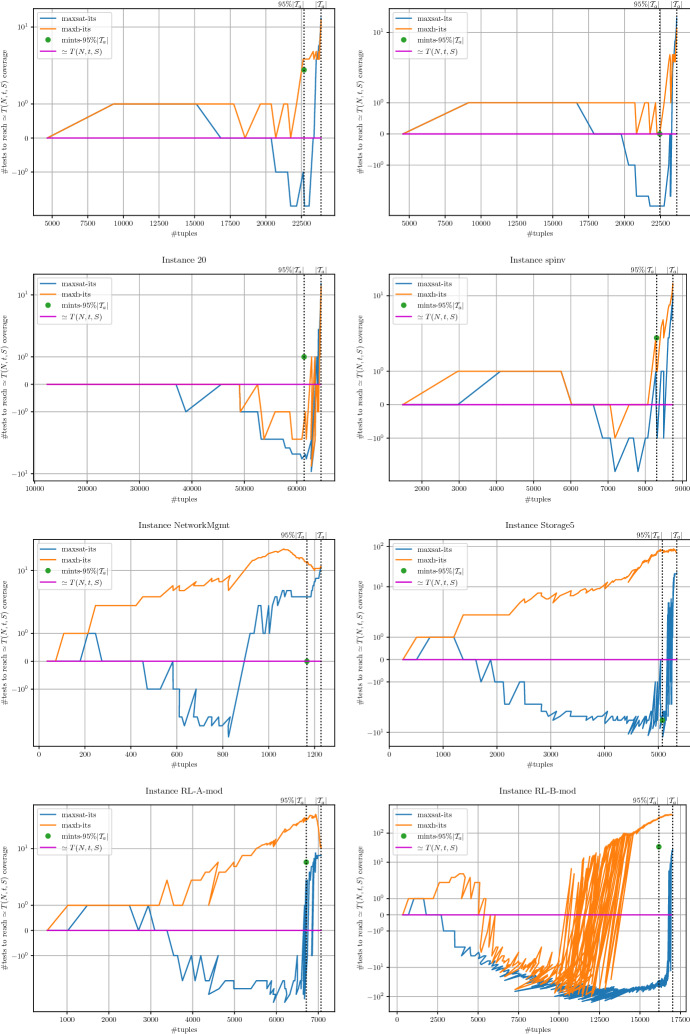
Comparison of the required number of tests for different methods with regards to the number of test used by $\simeq T(N,t,S)$ (as base) to cover each number of tuples

**Fig. 3:**
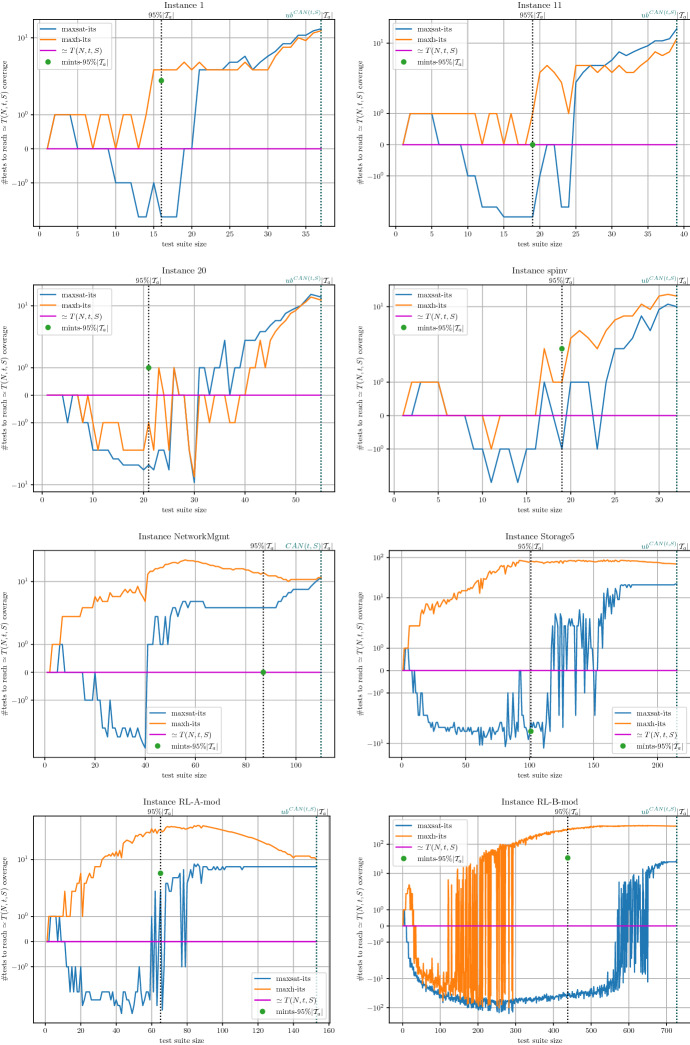
Comparison of the required number of tests for different methods to cover as much tuples at each test from $\simeq T(N,t,S)$ (as base)

**Fig. 4:**
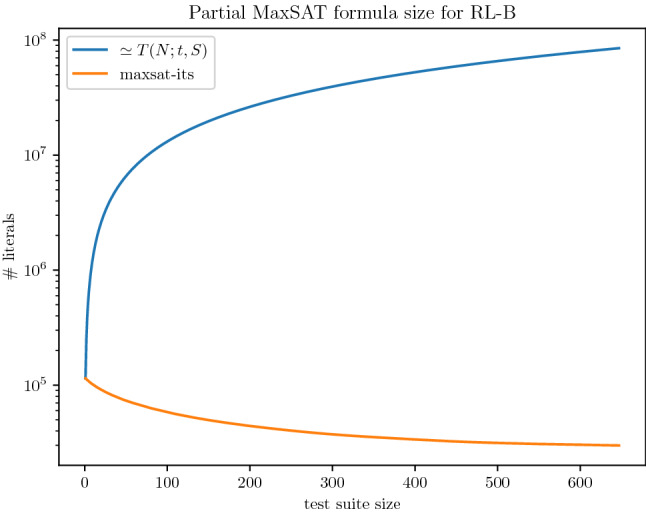
Partial MaxSAT formula size for RL-B in literals as a function of test suite size

**Results:** We present the relative performance of the previous four approaches respect to the best incomplete MaxSAT approach (*tt-open-wbo-inc*) for solving the Tuple Number problem from Sect. 11.2, referred as $\simeq T(N;t,S)$ (we use the symbol $\simeq$ to indicate that the values reported for $\simeq T(N;t,S)$ correspond to suboptimal solutions). All the approaches shown in this section also use the incomplete SAT-based MaxSAT solver *tt-open-wbo-inc*, except $sat-its$ which uses the Glucose41 SAT solver. For the encoding of equation (a) of *CCX* we use variation a.2 $(c^{i}_{\tau } \leftrightarrow c^{i-1}_{\tau } \vee x_{i,p,v})$ as in Sect. 11.2.

To perform a fair comparison we tried to execute all the algorithms within the same runtime conditions. We use as a reference the runtime that $maxsat-its$ needs to cover all the allowed tuples. In more detail, we set a timeout of 100s to each iteration of the $maxsat-its$ approach.^14^ Therefore, the total runtime in seconds consumed by $maxsat-its$ is the number of tests it reaches multiplied by 100. For $maxh-its$ and $sat-its$, the timeout is the total runtime consumed by $maxsat-its$. For $mints-95\%|\tau _a|$, we use as timeout the runtime consumed by $\simeq T(N;t,S)$ to reach 95% of coverage. Finally, for $\simeq T(N;t,S)$, we use a timeout of $N \cdot 100$ seconds for each *N*. Notice that in this last case we are ensuring that for a given *N*, both $\simeq T(N;t,S)$ and $maxsat-its$ approaches will have the same execution time limits.

All approaches have been executed with 3 seeds and the mean is reported. The experimental results are presented in Figs. 2 and 3. As in Sect. 11.2, we only plot the most representative instances.

Figure 2 shows the increment (or decrement) of the number of tests required by $maxsat-its$, $maxh-its$ and $mints-95\%|\tau _a|$ to cover the same number of tuples as $\simeq T(N;t,S)$. On the other hand, Fig. 3 shows the increment (or decrement) of tests required to reach the same coverage ratio as $\simeq T(N;t,S)$. For the $sat-its$ approach we found that in most cases it is able to cover only one tuple per test, so we decided to exclude these results in the figures as they were clearly outperformed by the rest of the presented approaches.

In both figures, we plot a vertical line to show the points where $\simeq T(N;t,S)$ reaches 95% and 100% of tuples covered.

In general, $maxsat-its$ clearly outperforms $maxh-its$. This can be expected since the nature of the incremental approach is to do the best at each possible iteration, and $maxsat-its$ tackles exactly this goal by solving the Tuple Number problem, while $maxh-its$ do not.

We also observe that $maxsat-its$ outperforms the tuple coverage that $\simeq T(N;t,S)$ can achieve on the first tests. Particularly, $maxsat-its$ is able to improve the number of tests required to cover 95% of the allowed tuples in 7 of the 8 instances we show in Figs. 2 and 3. On the other hand, above 95%, $\simeq T(N;t,S)$ seems to be the best approach in terms of using fewer tests for the same coverage. This makes sense since the incomplete nature of $maxsat-its$ makes it less efficient when approaching the complete coverage, which may not be needed for several applications.

In Fig. 2 we observe an erratic behaviour of instance *RL-B*, which is the largest instance that we had available. These results are in line with the ones in Fig. 1 of Sect. 11.2, and shows the possible issues that $\simeq T(N;t,S)$ can suffer when dealing with large instances. In particular, Fig. 4 shows the number of literals of the MaxSAT instance solved by $\simeq T(N;t,S)$ and $maxsat-its$ as the size of the test suite increases for the *RL-B* benchmark. We observe that $\simeq T(N;t,S)$ has to deal with an increasing size of the Partial MaxSAT instance proportional to the number of tests in the test suite. In contrast, for $maxsat-its$ the size of the instance decreases, since only one test is encoded and the number of tuples to cover decreases along with the size of the test suite built so far. This is an interesting insight since RL-B instance comes from an industrial application and it may reflect what we can face in harder real-world scenarios. Therefore, $maxsat-its$ may seem more well suited for these harder real-world domains and may extend the reach of Combinatorial Testing for more complex SUTs.

Finally, although $mints-95\%|\tau _a|$ is not consistently the best option to obtain a good suboptimal test suite that covers 95% of the total tuples, it obtains the best result on instances *NetworkMgmt* and *Storage5*. Moreover, it is the only method that guarantees optimality when combined with a complete MaxSAT solver.

## Conclusions

We have shown that MaxSAT technology is well-suited for solving the Covering Array Number problem for Mixed Covering Arrays with Constraints through SAT technology. In particular, we discussed efficient encodings and how MaxSAT algorithms perform on them.

We also presented MaxSAT encodings for the Tuple Number problem. To our best knowledge, this is the first time that this problem is studied with SUT Constraints. Additionally, we presented a new incomplete algorithm that can be applied efficiently to solve those instances where the Tuple Number problem encoding into MaxSAT is too large. In particular, we proved we can build good enough solutions by incrementally adding a new test synthesized through a MaxSAT query that aims to maximize the coverage of additional allowed tuples, respect to the test suite under construction.

Another interesting result that we obtained is that if we do not aim to cover all *t*-tuples but a *statistically significant* fraction, we can save a great number of tests. We experimentally showed that to cover a 95% percentage, we just need, on average, a 52% percentage of the best suboptimal solution reported so far. This is of high practical importance for applications where test cases are expensive according to the budget.

From the point of view of Combinatorial Testing, it is reasonable to say that the practical and theoretical interest application of our findings and approaches will grow proportionally to the hardness or complexity of the SUT constraints. This will certainly extend the reach of Combinatorial Testing to more challenging SUTs.

From the point of view of Constraint programming, the lessons learnt on how to design efficient encodings for MaxSAT solvers can be exported to solve similar problems. These problems are roughly characterized by having an objective function whose size is proportional to the best known upper bound.

SAT and MaxSAT communities will also benefit from new challenging benchmarks to test the new advances in the field. Moreover, any future advance in MaxSAT technology can be applied to solve more efficiently the Covering Array Number and Tuple Number problems with no additional cost.

## Footnotes

1 For $N \ge CAN(t,P)$, the Tuple Number problem essentially corresponds to determine the number of allowed tuples in the corresponding MCAC problem.

2 We replace $A \rightarrow B$ with $(\lnot A \vee B)$, and $A \leftrightarrow B$ with $(A \rightarrow B) \wedge (A \leftarrow B)$ where *A* and *B* are disjunctions of literals.

3 Notice that $\tau$ could be covered by other tests but the respective $c^{i}_{\tau }$ variables be False.

4 By *simulate*, we informally refer to perform the same sequence of upper bound refinements and the same filtering based on unit propagation.

5 $sat.model[b_i]$ is 1 if $b_i$ is assigned to True in the model, otherwise it is 0.

6 Recall that hard clauses have weight $\infty$.

7 In Ansótegui et al. (2012), the first call to $next\_stratum$ returns the cluster of all hard clauses since their representative weight is $\infty$

8 Notice that $(CNF(\sum _{b \in b\_vars} b = 1), \infty )$ is a set of clauses that have $\infty$ weight.

9 Notice that if $N \ge CAN(t,S)$, then $|\mathcal {T}_a| - T(N;t,S)$ is 0. However, we keep this expression in case we want to interchange $\mathcal {T}_a$ by $\mathcal {T}$. i.e., if we do not prefilter the forbidden tuples (see Remark 5).

10 We adapted RC2 MaxSAT solver to report suboptimal solutions when applying the stratified strategy (see Sect. 8)

11 Unfortunately *RC2* MaxSAT solver does not allow to specify a seed.

12 To the best of our knowledge this is the best known upper bound for $t=2$ for this instance.

13 The algorithm allows to add more than one test at a time, but this experiment is out of reach in this paper.

14 We assume that $maxsat-its$ is able to cover at least one more tuple in 100 seconds
